# Dominant-negative PSMB10 disrupts immunoproteasome assembly and leads to transient T lymphopenia

**DOI:** 10.70962/jhi.20250129

**Published:** 2026-04-09

**Authors:** Sujal Ghosh, Yuqin Wang, Christoph Schultheiss, Arndt Borkhardt, Angelique Sanchez Dafun, Liisa Knipp, Sebastian Marwitz, Stephan Ehl, Clara Soulard, Elie Haddad, Debayan Dey, Grace Evans, Zainab M. Golwala, Sabrina B. Bennstein, Hans-Jürgen Laws, Meino Rohlfs, Thomas Magg, Mascha Binder, Alexandra Y. Kreins, Marie-Pierre Bousquet, Julien Marcoux, Christoph Klein, Graeme L. Conn, Silke Meiners, Fabian Hauck

**Affiliations:** 1 Department of Pediatric Oncology, Hematology and Clinical Immunology, Medical Faculty, Heinrich-Heine-University, University Hospital Düsseldorf, Düsseldorf, Germany; 2 Immunology and Cell Biology, Research Center Borstel - Leibniz Lung Center, Airway Research Center North, Member of the German Center for Lung Research, Borstel, Germany; 3 Division of Medical Oncology, University Hospital Basel, Basel, Switzerland; 4 Laboratory of Translational Immuno-Oncology, Department of Biomedicine, University Hospital Basel, Basel, Switzerland; 5 Institut de Pharmacologie et de Biologie Structurale, Université de Toulouse, CNRS, Toulouse, France; 6 Infrastructure Nationale de Protéomique, ProFI, UAR 2048, Toulouse, France; 7 Histology, Research Center Borstel - Leibniz Lung Center, Airway Research Center North, Member of the German Center for Lung Research, Borstel, Germany; 8 Institute for Immunodeficiency, Center for Chronic Immunodeficiency, Faculty of Medicine, Medical Center, University of Freiburg, Freiburg, Germany; 9 Pediatric Immunology and Rheumatology Division, CHU Sainte-Justine, Department of Pediatrics, University of Montreal, Montreal, Quebec, Canada; 10 Department of Biochemistry, Emory University School of Medicine, Atlanta, GA, USA; 11 Infection, Immunity and Inflammation, Research & Teaching Department, Great Ormond Street Institute of Child Health, University College London, London, UK; 12 Department of Immunology and Gene Therapy, Great Ormond Street Hospital for Children NHS Foundation Trust, London, UK; 13 Institute for Transplantation Diagnostics and Cell Therapeutics, Medical Faculty, Heinrich-Heine University Düsseldorf, Düsseldorf, Germany; 14 Institute of Immunology, Faculty of Medicine, RWTH Aachen University, Aachen, Germany; 15 Department of Pediatrics, Dr. von Hauner Children’s Hospital, University Hospital, Ludwig-Maximilians-Universität München, Munich, Germany; 16 Institute of Experimental Medicine, Christian-Albrechts-University Kiel, Kiel, Germany

## Abstract

Immunoproteasomes are specialized multiprotein proteases that degrade intracellular proteins. Their products serve as peptides for human leukocyte antigen class I presentation playing a key role in antiviral defense and self-tolerance.

Monoallelic variants in immunoproteasome genes including proteasome subunit β type 10 have recently been associated with autoinflammatory diseases and severe combined immune deficiency. Their pathophysiological consequences remain poorly understood, and treatment options are scarce. We identified a newborn with severe T lymphopenia and a de novo dominant-negative PSMB10 p.G209R mutation after pathological T cell receptor excision circle newborn screening. We further applied molecular modeling, proteomics, transcriptomics, and ex vivo T lymphopoiesis. Simulations predicted, and biochemical studies confirmed, impaired immunoproteasome assembly and function leading to defective viral sensing and antigen presentation signatures in interferon-treated fibroblasts. Despite this, hematopoietic stem cells differentiated into T cells ex vivo, and the patient developed normal naïve T cell counts and a diverse T cell antigen receptor repertoire within the first year of life in contrast to all previously reported patients.

## Teaser

Severe neonatal T lymphopenia detected by newborn screening can reflect diverse underlying mechanisms. Ghosh et al. describe a monoallelic dominant-negative PSMB10 variant that impairs immunoproteasome assembly and is associated with transient neonatal T lymphopenia, highlighting a potentially dynamic and reversible form of early-life thymic dysfunction.

## Introduction

The degradation of intracellular proteins is largely mediated by the proteasome. The barrel-shaped 20S core of the standard proteasome consists of two outer α-rings and two inner β-rings. Three standard catalytic subunits, PSMB6 (β1), PSMB7 (β2), and PSMB5 (β5), confer acidic, basic, and hydrophobic proteolytic activity, respectively, and cleave proteins after the respective amino acids into small peptides for amino acid recycling and human leukocyte antigen (HLA) class I presentation (1). The immunoproteasome, which is expressed constitutively in immune cells and is induced upon inflammatory cytokine stimulation in nonimmune cells, exhibits altered peptide-cleavage properties to facilitate efficient antigen processing for presentation on HLA class I molecules (2). To achieve this, the catalytic standard subunits are exchanged by their inducible counterparts PSMB9 (β1i), PSMB10 (β2i), and PSMB8 (β5i) and assembled into the immunoproteasome (3, 4). In the thymus, a unique type of proteasome, the thymoproteasome, is located in cortical thymic epithelial cells (cTECs), consisting of the inducible PSMB9 and PSMB10, and the cTEC-specific PSMB11 (β5t) subunits (5). The thymoproteasome is crucial for positive T cell selection in the thymus (3, 6). In contrast, medullary TECs (mTECs) express immunoproteasomes and influence negative T cell selection (6).

Biallelic and monoallelic variants of the 20S core subunits, including the immunoproteasome, have been associated with proteasome-associated autoinflammatory syndromes (PRAAS) clinically characterized by type 1 interferon (IFN1) response–mediated autoinflammation (7). However, there is growing evidence that specific heterozygous variants located in the immunoproteasome subunit PSMB10 predominantly lead to systemic autoinflammatory diseases, and severe combined immune deficiency (SCID) partially manifesting as Omenn syndrome (8, 9, 10, 11).

SCIDs are inborn errors of immunity (IEIs) that can be diagnosed following the Primary Immune Deficiency Treatment Consortium definitions such as (a) very low T cell numbers (<0.05 × 10^9^/L), (b) pathogenic gene variant(s), (c) undetectable or low T cell receptor excision circles (TRECs) upon newborn screening or <20% of naïve CD4 T cells, and (d) transplacentally acquired maternal T cell engraftment (12). SCIDs are caused by genetic germline variants that intrinsically impair T cell development and/or function and are thus hematopoietic disorders (13). Inborn errors of thymic stromal cell development and/or function associated with congenital athymia also result in a SCID-like phenotype, with selective T lymphopenia and frequent Omenn syndrome (OS)-like features (14).

We here studied an infant with severe neonatal T lymphopenia identified by TREC-based newborn screening who carried a monoallelic de novo PSMB10 p.G209R private variant. We provide detailed mass spectrometry (MS) and biochemical evidence that this specific PSMB10 variant impairs immunoproteasome assembly consistent with a dominant-negative (DN) effect. The clinical and experimental findings support a model in which altered proteasome assembly contributes potentially to an extra-hematopoietic defect, which affects the thymic microenvironment and is associated with transient neonatal T lymphopenia.

## Results

### TREC-based newborn screening identifies a case of severe neonatal T lymphopenia

**Figure 1.**
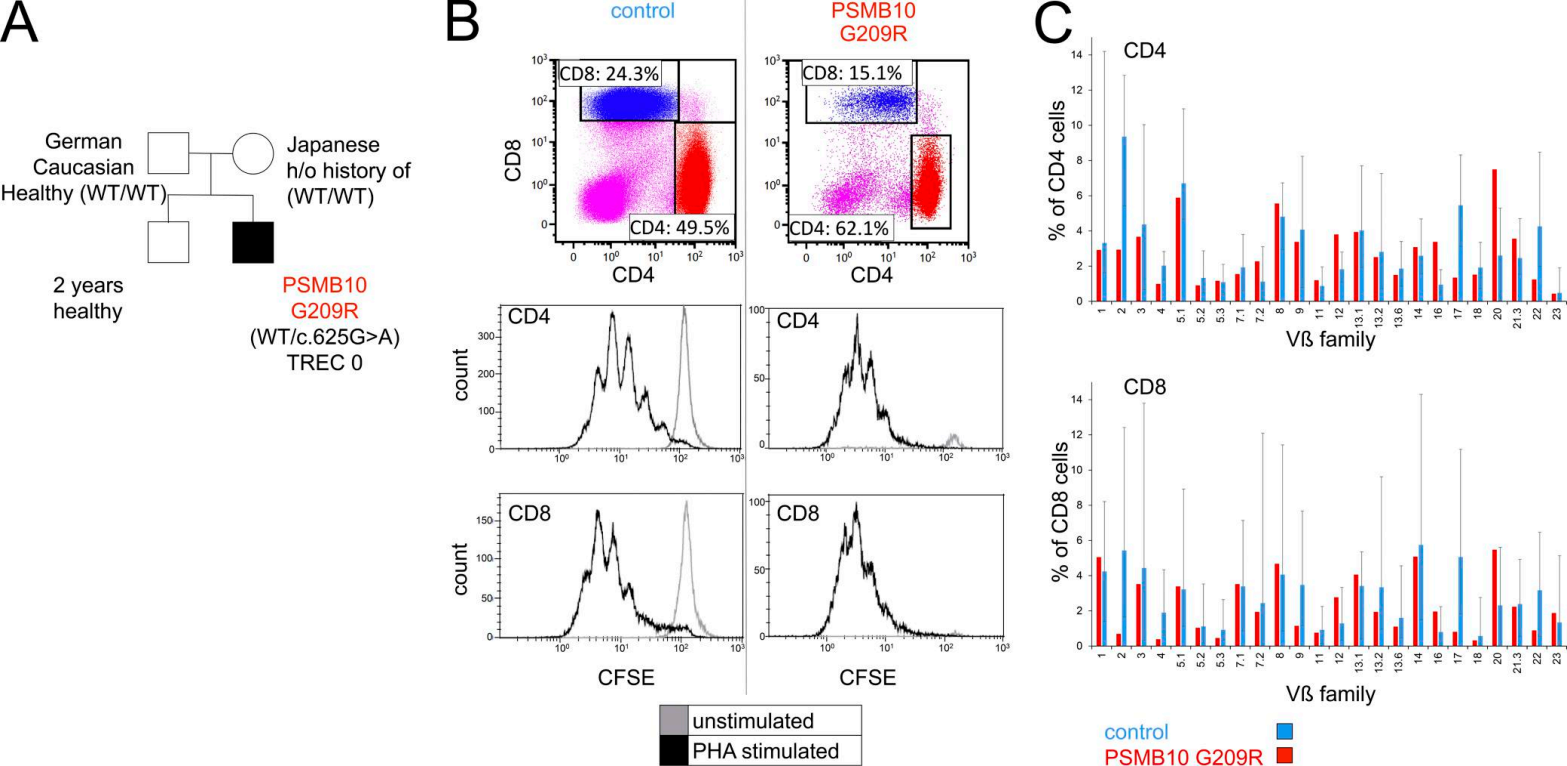
**TREC newborn screening identifies a case of severe neonatal polyclonal T lymphopenia. (A)** Genealogical tree of the index case (black box) with severe neonatal lymphopenia and his immunologically unaffected parents and siblings (light boxes and circles). **(B)** Flow cytometric dot plots (upper lane) of CD4 and CD8 T cells including their respective percentages of total lymphocytes and histogram plots (middle and lower lanes) of CFSE dilution in unstimulated (gray histogram) and PHA-stimulated (black histogram) CD4 and CD8 T cells of a control (left column) and the index patient (right column); *n* = 1. **(C)** Flow cytometry–based bar graphs of CD4 and CD8 TCR Vβ clonotypes (x axis) and their relative abundance (y axis) of a control (blue boxes) and the index patient (red boxes); *n* = 1 (15).

The index patient was the offspring of a nonconsanguineous German father and a Japanese mother (Fig. 1 A) who came to medical attention because of abnormal TREC newborn screening without any clinical phenotype. The mother was suffering from autoimmune thyroiditis but had no treatment during pregnancy. Otherwise, the family history was unremarkable; a 2-year older brother was healthy.

The patient’s immunological workup confirmed severe neonatal T lymphopenia. B cell and natural killer (NK) cell numbers were normal (Table 1). T cell proliferation after mitogenic (phytohemagglutinin [PHA]) (Fig. 1 B) and anti-CD3/CD28 stimulation (not shown) was normal. Despite severe neonatal T lymphopenia, a relevant fraction of the remaining CD4^+^ T cells displayed a naïve phenotype (28.3%) (Table 1) and a polyclonal T cell antigen receptor (TCR) Vβ repertoire by flow cytometry (Fig. 1 C). Immunoglobulin (Ig) M, IgG, IgA, and IgE levels were normal (Table 1).

**Table 1.** Immunological parameters of the monoallelic PSMB10 G209R patient over time

​	1 wk	13 mo	25 mo	38 mo	51 mo	66 mo
White blood cells, cells/µl	**5,818** (7,540–14,910)	8,324 (6,510–13,940)	9,333 (5,420–11,870)	10,228 (5,420–11,870)	8,300 (5,420–11,870)	7,277 (5,420–11,870)
Lymphocytes, cells/µl	**1,923** (3,500–13,100)	3,578 (2,600–10,400)	3,939 (2,700–11,900)	2,139 (1,700–6,900)	3,184 (1,700–6,900)	2,453 (1,100–5,900)
Neutrophils, cells/µl	**1,086** (1,960–4,710)	2,965 (1,400–4,550)	3,555 (1,790–5,640)	6,995 (1,790–5,640)	3,121 (1,790–5,640)	3,738 (1,790–5,640)
Eosinophils, cells/µl	254 (20–720)	**1,034** (60–430)	**526** (70–500)	226 (70–500)	**817** (70–500)	263 (70–500)
Monocytes, cells/µl	596 (570–1,900)	**429** (490–1,170)	**399** (440–950)	574 (440–950)	460 (440–950)	**391** (440–950)
Total T cells (CD3), cells/µl	**208** (2,300–7,000)	**919** (1,600–6,700)	**1,367** (1,400–8,000)	903 (900–4,500)	1,676 (900–4,500)	1,261 (700–4,200)
T helper cells (CD4), cells/µl	**134** (1,700–5,300)	**603** (1,000–4,600)	957 (900–5,500)	612 (500–2,400)	984 (500–2,400)	721 (300–2,000)
Naïve CD4 T cells (CD45RA), cells/µl	*38*	*253*	*428*	*262*	*401*	275
Naïve CD4 T cells (CD45RA), %	**28.3** (81.0–91.5)	**41.9** (75.8–88.1)	**44.7** (61.8–85.0)	**42.8** (61.8–85.0)	**40.7** (61.8–85.0)	**38.2** (61.8–85.0)
Memory CD4 T cells (CD45RO), cells/µl	*92*	*281*	*395*	*306*	*488*	465
Memory CD4 T cells (CD45RO), %	**68.6** (5.1–11.4)	**46.6** (10.0–15.6)	**41.3** (14.8–37.2)	**50.0** (14.8–37.2)	**49.6** (14.8–37.2)	**64.5** (14.8–37.2)
Cytotoxic T cells (CD8), cells/µl	**18** (400–1,700)	**149** (400–2,100)	**262** (400–2,300)	**151** (300–1,600)	427 (300–1,600)	435 (300–1,800)
Naïve CD8 T cells (CD45RA), cells/µl	*14*	*75*	*124*	*79*	*237*	214
Memory CD8 T cells (CD45RO), cells/µl	*4*	*68*	*110*	*82*	*223*	216
CD4/CD8 ratio	*7:1*	*4:1*	*3,6:1*	*4:1*	*2,3:1*	1,7:1
TCRab T cells, cells/µl	*138*	*719*	*1,235*	*797*	*1,394*	1,140
TCRgd T cells, cells/μl	*5*	*115*	*121*	*73*	*192*	148
TRECS/10^6^ T cells	​	​	​	​	*1.988*	​
NK cells (CD3^−^CD56^+^CD16^+^), cells/µl	254 (200–1,400)	865 (200–1,200)	632 (100–1,400)	285 (100–1,000)	267 (100–1,000)	492 (90–900)
Total B cells (CD20), cells/µl	**423** (600–1,900)	1,822 (773–1,990)	1,832 (529–1,930)	631 (529–1,930)	1,141 (323–1,000)	512 (323–1,000)
Naïve B cells (IgD^+^CD27^−^), cells/µl	*399*	1,691 (574–2,215)	1,602 (419–1,748)	504 (419–1,748)	829 (245–799)	343 (245–799)
Intermediate B cells (IgD^+^CD27^+^), cells/µl	*7*	67 (43–118)	78 (39–208)	45 (39–208)	155 (29–113)	51 (29–113)
Memory B cells (IgD^−^CD27^+^), cells/μl	*1*	34 (13–54)	99 (20–115)	62 (20–115)	126 (18–94)	96 (18–94)
IgM (mg/dl)	40 (3–32)	66 (14–82)	165 (36–144)	134 (36–144)	183 (36–144)	189 (36–144)
IgG (mg/dl)	986 (390–1,050)	610 (470–1,230)	776 (540–1,340)	818 (540–1,340)	904 (590–1,430)	888 (590–1,430)
IgA (mg/dl)	<5 (<14)	28 (<14)	54 (<80)	81 (11–142)	92 (11–142)	106 (11–142)
IgE (U/ml)	**22** (<2)	26 (<60)	12 (<60)	14 (<60)	11 (<60)	n.d.
Vaccine serology inactivated	n.d.	T+D+HepB+	T+D+HepB+	T+D+HepB+	T+D+HepB+	T+D+HepB+
Live	​	​	M+Mu+R+V+	M+Mu+R+V+	M+Mu+R+V+	M+Mu+R+V+

Reference intervals according to (16) white blood cells, neutrophils, eosinophils, monocytes (17), and lymphocytes; total T, CD4 helper, cytotoxic CD8, and NK and B cells (18); naïve and memory CD4 percentages; and (19) B cell subpopulations and (20) TREC levels. Reduced cell counts are indicated in bold. Cell counts without reference ranges are indicated in italics. Ig reference intervals according to the local standard (University Hospital Duesseldorf). Abbreviations: T: tetanus; D: diphtheria; Hep B: hepatitis B; M: measles; Mu: mumps; R: rubella; V: varicella.

Known causes of secondary T lymphopenia were ruled out, and therefore, an IEI with a T^−^B^+^NK^+^ immunophenotype was suspected. Clinically, the patient was well, without any signs of infection, autoinflammation, or autoimmunity at this point in time. The patient was taken care of in homely isolation, was nourished with infant formula as the mother was cytomegalovirus (CMV)-positive, and was put on prophylactic cotrimoxazole, fluconazole, and Ig replacement therapy.

### A de novo monoallelic PSMB10 p.G209R private variant is associated with severe neonatal T lymphopenia

**Figure 2.**
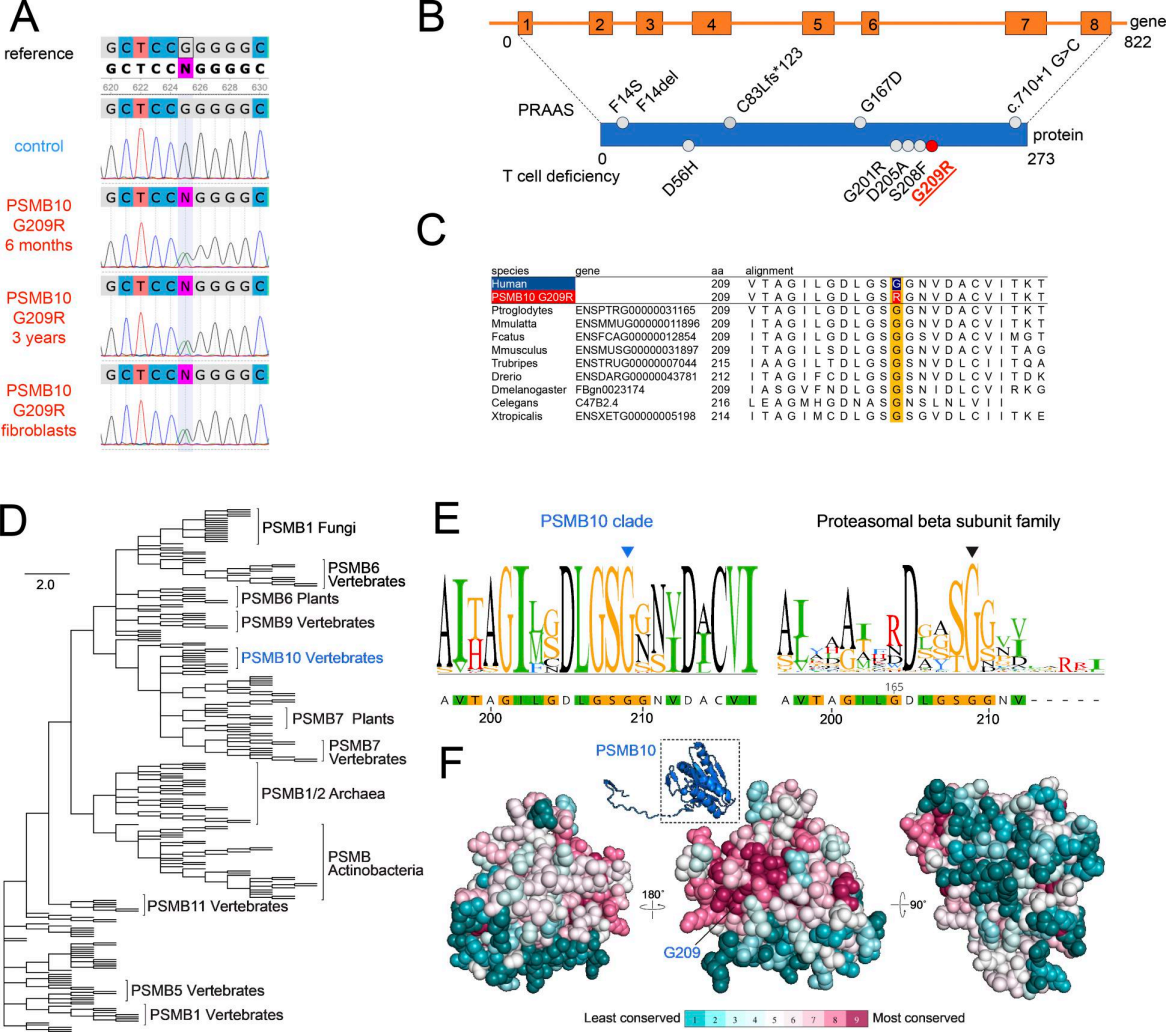
**De novo monoallelic PSMB10 p.G209R private variant is highly conserved and located in an invariant surrounding protein surface. (A)** Alignment of the PSMB10 reference sequence (ENST00000358514) with Sanger sequencing electropherograms of PB from a control, PB from the index patient at 6 mo and 3 years of age, and fibroblasts of the index patient. **(B)** Scheme of the *PSMB10* gene with exons (boxes) and introns (beams) and the PSMB10 protein. Pathogenic PSMB10 protein variants reported to cause PRAAS and T lymphopenia are depicted above and below the protein scheme, respectively (not drawn to scale). **(C)** Multiple sequence alignment of human PSMB10 WT (top row) and PSMB10 G209R (second top row) and its homologs ranging from chimpanzee to frog are shown. **(D)** Proteasomal β subunit peptidase family ML-based phylogenetic tree indicating separation of paralogs of PSMB10, PSMB7, and others as indicated. The vertebral PSMB10 clade (blue) is part of a larger vertebrate and plant PSMB7 group. **(E)** PSMB10 sequence and residue conservation LOGO plot surrounding G209 for the PSMB10 clade only (left) and the entire β subunit peptidase family (right) with G209 indicated with an arrowhead. **(F)** Three rotated views of the PSMB10 structure with residue conservation within the PSMB10 clade mapped on its surface. The cartoon thumbnail image and box indicate the region of PSMB10 shown and the orientation of the center view. Amino acid conservation is displayed with a color gradient as indicated below the structure of cyan (least conserved) to purple (most conserved). The residues surrounding G209 form the most highly conserved region of the protein surface. PB, peripheral blood.

To determine the genetic etiology of the patient’s selective T lymphopenia, trio whole-exome sequencing was performed. No known SCID-causing variant was identified, but a de novo monoallelic missense variant in PSMB10 (ENST00000358514: c.625G>A; p.G209R) was considered as a candidate gene defect. The monoallelic variant was confirmed by Sanger sequencing in peripheral blood mononuclear cells (PBMCs) at 6 mo and 3 years after birth, as well as in fibroblasts (Fig. 2 A). The variant has not been reported in the gnomAD, ExAC, ClinVar, or 1,000 Genomes Project databases as mono- nor biallelic and thus can be considered a private variant.

The substituted amino acid is located at a highly conserved position (Fig. 2, B and C), and therefore, several in silico pathogenicity scores rated it as probably damaging combined annotation-dependent depletion ([CADD] 33, PolyPhen [21, 22]), deleterious sorting intolerenat from tolerant ([SIFT] [23]), and disease-causing (MutationTaster [24]).

Importantly, a murine Psmb10 G170W variant that is homologous to the human PSMB10 G209R has been reported in a mouse model (25) where it caused systemic autoinflammation and SCID in the biallelic state and selective T lymphopenia in the monoallelic state.

### PSMB10 G209 is highly conserved and located in an invariant protein surface

To further validate PSMB10 p.G209R as a novel cause of severe neonatal T lymphopenia, we conducted a phylogenetic study of the complete β subunit peptidase family using the maximum-likelihood (ML) method (Fig. 2 D). This analysis separated paralogs of this family from vertebrates, as well as homologs in plants, Archaea, and Actinobacteria. The phylogenetic tree closely grouped β subunits PSMB6, PSMB7, PSMB9, and PSMB10, with fungal PSMB1 also sharing close similarity to vertebrate PSMB6. The phylogenetic tree grouped PSMB10 sequences as a single clade within a larger group with vertebrate and plant PSMB7 sequences (Fig. 2 D).

Extracting sequences from the PSMB10 clade for multiple sequence alignment–based conservation analysis revealed that the position corresponding to G209 and surrounding residues was highly conserved in the PSMB10 clade (Fig. 2 E). This high conservation was consistent with an important role for this PSMB10 region in the interaction with PSMB1 in the immunoproteasome complex. G209 was also highly conserved in the entire β subunit peptidase family (Fig. 2 E) further suggesting its importance in proteasome complex formation. Residue conservation scores were mapped onto the PSMB10 structure (from PDB 6E5B) using ConSurf, revealing high localized conservation on the protein surface surrounding G209 (Fig. 2 F).

### PSMB10 G209R results in assembly of a less stable immunoproteasome in silico

**Figure 3.**
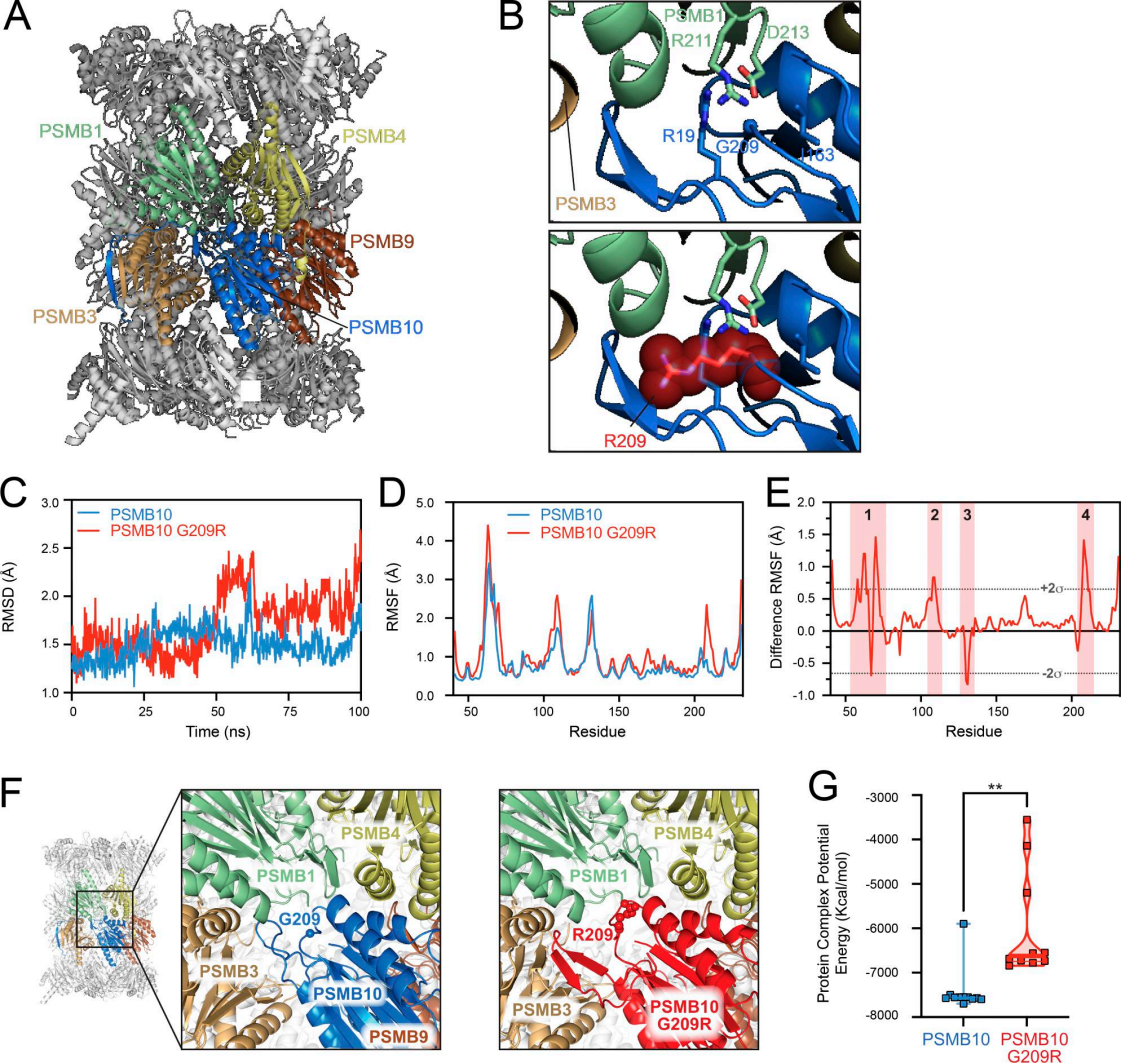
**PSMB10 G209R results in a less stable immunoproteasome assembly in silico. (A)** 20S immunoproteasome structure (PDB 6E5B) highlighting PSMB10 (in blue) and neighboring β subunits PSMB1 (green), PSMB3 (light brown), PSMB4 (yellow), and PSMB9 (dark brown) of the inner rings of the assembled immunoproteasome. **(B)** Zoomed views of the immunoproteasome subunits surrounding PSMB10 residue G209 (upper panel) and G209R (lower panel) suggesting major clashes with R19 within PSMB10 and R211 and D213 of PSMB1. **(C)** Overall protein RMSD over the 100-ns MDS production run for PSMB10 WT (blue) and PSMB10 G209R (red). **(D and E)** (D) Residue backbone atom RMSF and (E) difference RMSF (PSMB10 G209R minus PSMB10 WT at each residue). The dotted lines denote two standard deviations (±2σ) from the average difference RMSF, and residues outside these values were considered to have the most significant changes in residue dynamics and corresponded to four protein regions (1–4, red shading). **(F)** Protein–protein docking of representative structures of PSMB10 WT (left, zoomed-in view) is accomplished with minimal clashes with its neighboring proteins. In contrast, PSMB10 G209R docking results in severe clashes with PSMB3 and PSMB1 (right, zoomed-in view). **(G)** Potential energy calculation for the 10 docked complexes for each protein reveals significantly reduced complex stability with PSMB10 G209R resulting from the identified steric clashes with neighboring subunits (*t* test, **P < 0.002).

To understand the effects of PSMB10 G209R on the structure of the assembled immunoproteasome, we performed in silico modeling, molecular dynamics simulation (MDS), and docking calculation. The immunoproteasome shares the same overall architecture as the standard proteasome (26), but PSMB7 is replaced by the immunoproteasome-specific subunit PSMB10 (Fig. 3 A).

G209 in PSMB10 is surrounded by R19 from the same protein, and R211 and D213 from the neighboring PSMB1 (Fig. 3 B). An in silico G209R substitution in PSMB10 (from PDB 6E5B) resulted in an extensive clash with R19 and residues 25–28 in PSMB10 and with the R211, D213, and residues 184–187 in the neighboring PSMB1 (Fig. 3 B). However, the PSMB10 residue G209 was located in a loop that might allow for more extensive reorganization to relieve these local clashes and still accommodate the G209R substitution. Therefore, to examine how the PSMB10 G209R subunit impacts on the structure and interactions with other immunoproteasome subunits, we investigated the flexibility and conformation of PSMB10 WT and PSMB10 G209R subunits using MDS. Root mean square deviation (RMSD) analysis revealed more extensive structural deviation and overall flexibility associated with PSMB10 G209R (Fig. 3 C). Root mean square fluctuation (RMSF) identified the most flexible regions in both PSMB10 WT and PSMB10 G209R (Fig. 3 D), and difference RMSF mapped four regions of most significant change in dynamics (Fig. 3 E): (1) multiple residues within the region 55–70; (2) residues 108–109; (3) residues 130–131; and (4) residues 207–210 containing the site of the G209R substitution.

Thus, the simulations of PSMB10 WT and PSMB10 G209R proteoforms confirmed an increase in flexibility in the region surrounding the amino acid substitution, which could interfere with its assembly into the immunoproteasome. Further, regions 1 and 3 were in proximity in PSMB10 and their coordinated increase in dynamics in PSMB10 G209R strongly suggests that they influence each other’s conformation, thereby altering interactions within the assembled immunoproteasome.

To determine how increased flexibility associated with the G209R substitution altered PSMB10 interactions within the assembling immunoproteasome, PSMB10 WT and PSMB10 G209R were docked with an assembly of neighboring subunits with full flexibility allowed for all proteins near the docking interface (Fig. 3 F). This analysis revealed low potential energy for the overall complex for the majority of PSMB10 WT structures but significantly higher potential energy for the PSMB10 G209R (Fig. 3 G). Specifically, the G209R substitution altered the structure and dynamics of two PSMB10 loops (regions 1 and 3), resulting in reduced favorable interactions and increased steric clashes with both the PSMB3 and PSMB1 subunits.

### PSMB10 G209R alters immunoproteasome activity in skin fibroblasts

**Figure 4.**
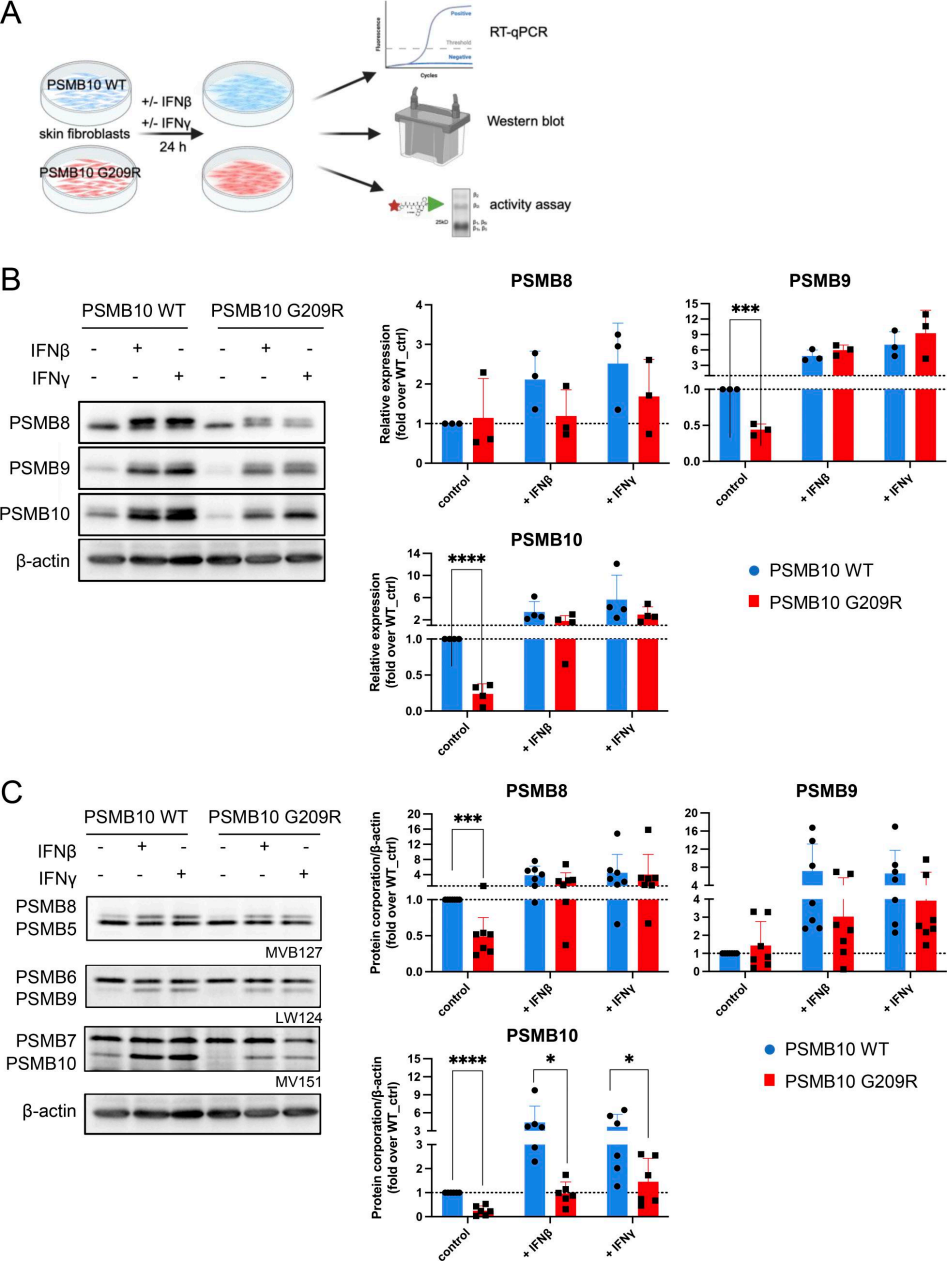
**Reduced immunoproteasome activity in IFN-stimulated PSMB10 G209R skin fibroblasts. (A)** Primary neonatal skin fibroblasts isolated from the patient with the PSMB10 G209R mutation and age-matched neonatal primary PSMB10 WT skin fibroblasts were treated with 75 U/ml IFNγ or 100 U/ml IFNβ using cells from passages 4–10 for RNA, protein, and activity analyses (scheme was prepared using BioRender). **(B)** Western blot analysis of immunoproteasome subunits PSMB8-10 with β-actin as a loading control. The two bands for PSMB10 represent the pro- and the mature forms. Upon IFN stimulation, expression and integration of PSMB10 into an active 20S proteasome are enhanced as evidenced by the presence of mainly mature PSMB10, which is generated upon assembly into the 20S and proteolytic removal of its propeptide. Representative western blot is shown, and densitometric analysis of 3–4 independent experiments where relative expression of PSMB8, PSMB9, and PSMB10 to β-actin was normalized to the PSMB10 WT control (multiple unpaired *t* test, P value: *** <0.001, **** <0.0001). **(C)** ABP analysis of proteasome activity using the pan-reactive probe MV151, PSMB6- and PSMB9-specific probe LW124, and PSMB5- and PSMB8-specific probe MVB127 (*n* = 6–7, multiple unpaired *t* test, P value: * <0.05, *** <0.001, **** <0.0001). The β-actin loading controls shown in B and C and Fig. S1 B are identical, as these panels were derived from the same experiment and immunoblot. Source data are available for this figure: SourceData F4.

To investigate the predicted destabilization upon incorporation of PSMB10 G209R into the immunoproteasome and its functional consequences, we analyzed primary skin fibroblasts obtained from the patient and compared them with healthy newborn PSMB10 WT skin fibroblasts. Skin fibroblasts express only minor baseline amounts of immunoproteasome subunits. We therefore induced immunoproteasome expression and formation by stimulating the cells with either IFN1 such as IFNβ or IFN2 such as IFNγ for 24 h to monitor both RNA levels and formation of immunoproteasomes (Fig. 4 A).

**Figure S1.**
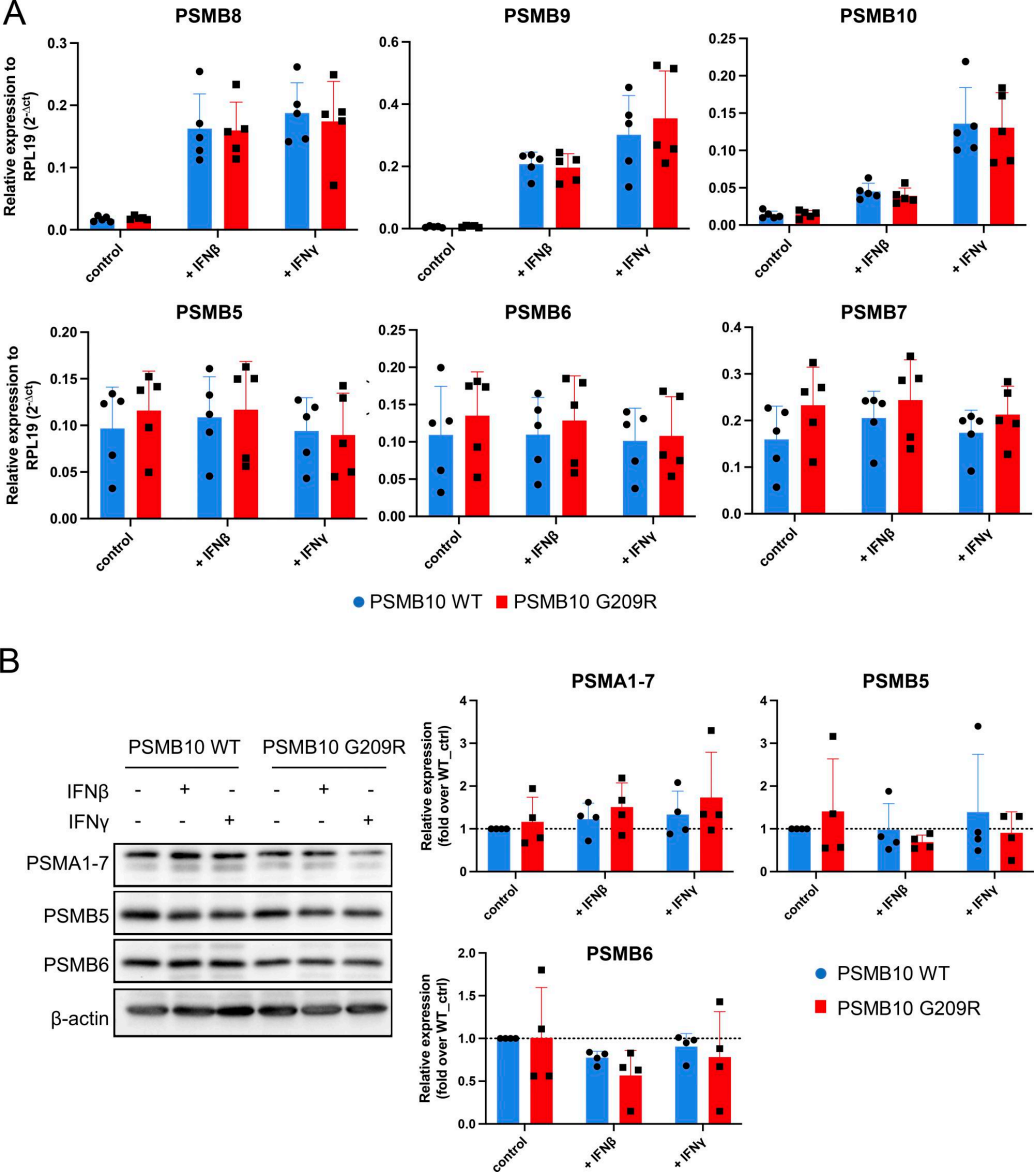
**RNA and protein analysis of immuno- and standard proteasome subunits in PSMB10 WT and PSMB10 G209R skin fibroblasts. (A)** RT-qPCR analysis of immunoproteasome subunits PSMB8-10 and the standard subunits PSMB5-7 with normalization to RPL19 (2^-delta CT^) upon stimulation with 75 U/ml IFNγ or 100 U/ml IFNβ for 24 h (*n* = 5, multiple unpaired *t* test). **(B)** Western blot analysis of proteasome subunits PSMA1-7, PSMB5, and PSMB6 with β-actin as the loading control (*n* = 3, multiple unpaired *t* test). The β-actin loading controls shown in B and Fig. 4, B and C are identical, as these panels were derived from the same experiment and immunoblot. Source data are available for this figure: SourceData FS1.

Stimulation with IFNβ or IFNγ resulted in pronounced transcriptional upregulation of all three catalytic immunoproteasome subunits PSMB8, PSMB9, and PSMB10 (Fig. 4 B). We did not observe significant differences in the RNA levels of all three immunoproteasome subunits between PSMB10 WT and PSMB10 G209R cells neither at baseline nor upon IFN treatment (Fig. S1 A). RNA expression of the corresponding standard subunits PSMB5, PSMB6, PSMB7 was not regulated by IFNs (Fig. S1 A). Baseline protein expression of PSMB9 and PSMB10 was significantly reduced in PSMB10 G209R skin fibroblasts compared with WT controls (Fig. 4 B), suggesting reduced protein stability as RNA levels were not altered. We noted that the induction of the immunoproteasome subunits was slightly reduced in the mutant cells, which was lost when normalized to the respective untreated controls. The two bands for PSMB10 represent the pro- and the mature forms. Upon IFN stimulation, expression and integration of PSMB10 into an active 20S proteasome are enhanced as evidenced by the presence of mainly mature PSMB10 upon assembly into the 20S and proteolytic removal of its propeptide. Constitutively expressed α subunits of the 20S proteasome (PSMA1-7) and the noninducible standard catalytic subunits PSMB5 and PSMB6 were not altered neither by IFN treatment nor in mutant cells (Fig. S1 B).

Next, we determined the assembly of the immunoproteasome subunits into a mature and active proteasome. We made use of activity-based probes (ABPs), which covalently bind to the active sites and thereby label them (27, 28). These ABPs thus only label active subunits that are incorporated into the mature and fully assembled 20S proteasome. The number of active sites can be determined upon electrophoretic resolution of the fluorescently labeled subunits according to their molecular weight. We used a set of three ABPs: MV151 labels all active sites but was used here to determine the activity of PSMB7 and PSMB10 as the other catalytic subunits cannot be properly separated due to overlapping molecular weights; LW124 binds specifically to PSMB6 and PSMB9; and MVB127 is specific for the PSMB5 and PSMB8 subunits. Our activity analysis revealed impaired formation of active immunoproteasomes already at baseline in PSMB10 G209R skin fibroblasts compared with the WT controls with signals for PSMB8 and PSMB10 being significantly reduced (Fig. 4 C). Upon treatment with IFNβ or IFNγ, formation of the immunoproteasome subunits was rapidly induced, but its activity was significantly impaired in PSMB10 G209R cells (Fig. 4 C).

### PSMB10 G209R alters immunoproteasome assembly in skin fibroblasts

**Figure 5.**
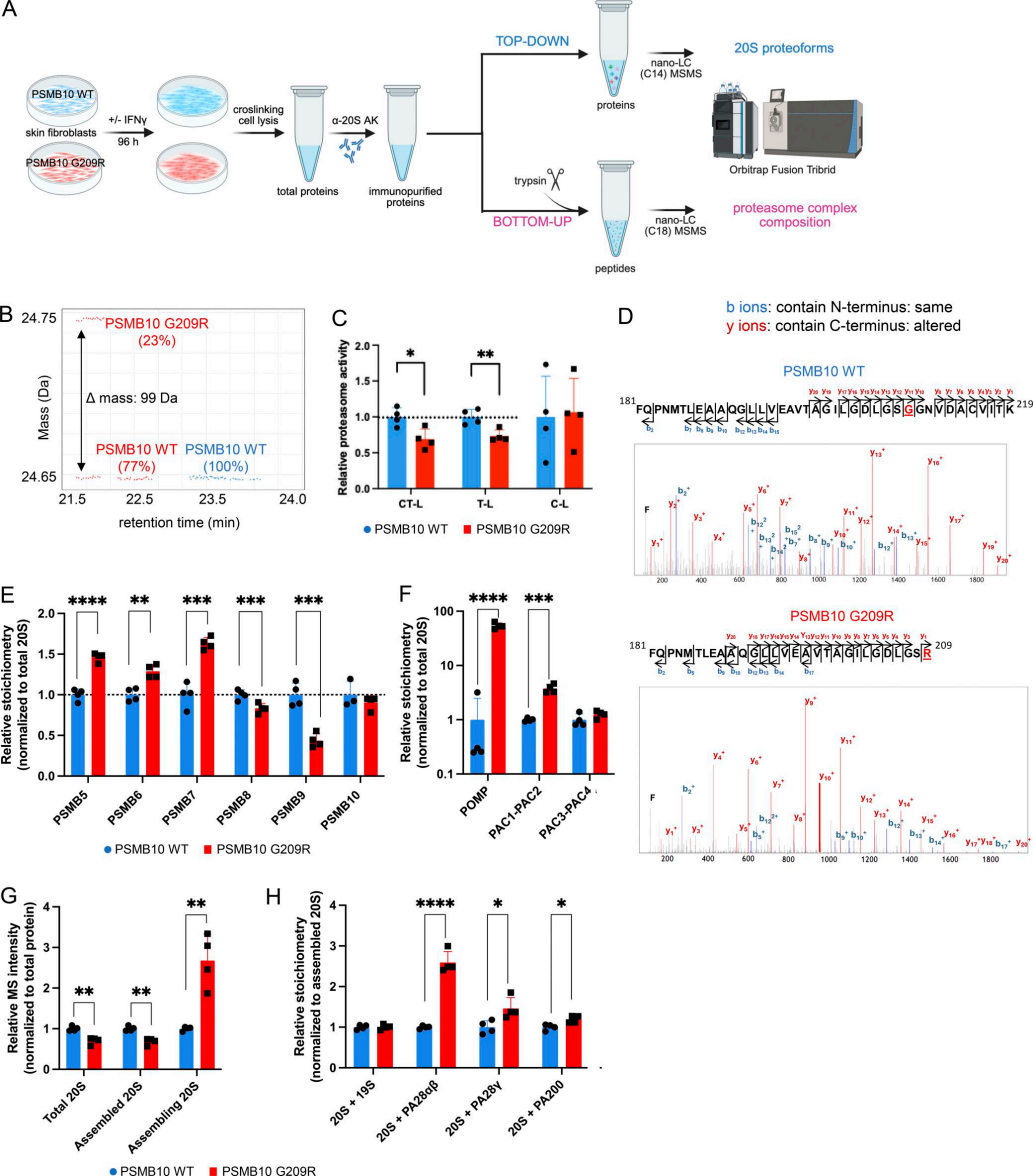
**Defective immunoproteasome assembly in IFN-stimulated PSMB10 G209R skin fibroblasts. (A)** Primary neonatal skin fibroblasts isolated from the patient with the PSMB10 G209R mutation and age-matched neonatal primary PSMB10 WT skin fibroblasts were treated with 75 U/ml IFNγ for 98 h for in-depth MS analysis of purified proteasome complexes (scheme was prepared using BioRender). **(B)** TD-MS map (using VisioProtMS) of immunopurified 20S from cross-linked IFNγ-treated PSMB10 G209R cells showing 77% incorporated PSMB10 WT [amino acid sequence 40–273] and 23% PSMB10 G209R [amino acid sequence 40–273] proteoforms at 24.65 and 24.75 Da, respectively, while PSMB10 WT cells contain only the WT form. The 99 Da increase in the mass corresponds to the G-to-R mutation. **(C)** Relative proteasome activities in cell lysates of IFNγ-treated cells using specific AMC-fluorescently labeled substrates for the CT-L, T-L, and C-L activities (*n* = 4, statistics based on *t* test, P value: * <0.05, ** <0.01). **(D)** BU-MS/MS analysis of immunopurified 20S from cross-linked IFNγ-treated PSMB10 WT and PSMB10 G209R skin fibroblasts showing the identified PSMB10 peptides corresponding to the amino acid sequence [181–219] for PSMB10 WT (top) and [181–209] for PSMB10 G209R (bottom) matching the MS/MS spectra with the identified b (in blue) and y (in red) fragment ions. **(E–H)** BU-MS/MS analysis of immunopurified 20S from PSMB10 WT and PSMB10 G209R cells showing the relative stoichiometries of (E) the 20S catalytic subunits, (F) the 20S-bound chaperones after normalization of MS intensities to the total amount of 20S noncatalytic subunits, (G) the relative MS intensities of total 20S, assembled 20S, and assembling 20S, which were estimated thanks to the stoichiometry of the 20S-bound PAC1-PAC2, and (H) the 20S-bound activators (*n* = 4, statistics based on *t* test, P value: * <0.05, ** <0.01, *** <0.001, **** <0.0001). CT-L activity, chymotrypsin-like activity; T-L activity, trypsin-like activity; C-L activity, caspase-like activity.

We interrogated assembly of the immunoproteasome in PSMB10 G209R cells by applying advanced MS analysis. PSMB10 WT and PSMB10 G209R skin fibroblasts containing two WT and one mutated and one WT allele, respectively, were stimulated with IFNγ for four days to allow for full induction and assembly of the immunoproteasome (Fig. 5 A). Subsequently, proteasome complexes were immunoprecipitated from the cells and analyzed via top-down MS (TD-MS). TD-MS involves MS analysis of proteins of interest without any prior digestion into peptides and thus provides their accurate molecular weight composition (Fig. 5 A) (29, 30). For immunopurification, we used an antibody directed against the α2 subunit of the 20S catalytic core (PSMA2) to copurify all subunits that are associated with fully and partially assembled 20S proteasomes (3). Semiquantitative MS analysis revealed that the PSMB10 G209R mutant and WT proteoforms accounted for ∼23 and 77% of the isolated subunits, respectively, as determined by a mass shift of 99 Da (Fig. 5 B). This is significantly lower than values usually obtained for mutations that do not alter proteasome assembly such as PSMB8 T75M (29). Since both assembling and assembled 20S proteasome can be immunopurified with the anti-α2 antibody, we next asked whether the mutation could stall immunoproteasome assembly. Of note, we observed reduced catalytic activity of the 20S complexes purified from IFNγ-stimulated PSMB10 G209R skin fibroblasts. Specifically, both the chymotrypsin-like and trypsin-like activities were significantly diminished compared with their WT controls, while the caspase-like activity was not altered (Fig. 5 C).

Using bottom-up (BU) MS with tryptic digestion of immunopurified 20S complexes (Fig. 5 A), we detected a distinct shorter tryptic peptide (amino acids 181–209) only in the PSMB10 G209R cells, since the G209R variant introduced a new tryptic cleavage site (Fig. 5 D). The longer peptide (amino acids 181–219) that is specific of the PSMB10 WT proteoform was detected both in the WT and in the mutant cells, confirming that both WT and G209R proteoforms have been copurified with the anti-α2 antibody in the heterozygous PSMB10 G209R fibroblasts.

PSMB10 G209R skin fibroblast proteasome populations also incorporated significantly less of the two other immunocatalytic subunits, PSMB8 and PSMB9, compared with WT cells, whereas the total stoichiometry of the PSMB10 protein (quantified using the MS signals of common peptides of the G209R mutant and WT proteoforms) was not changed overall (Fig. 5 E). We also noticed elevated copurification of several assembly chaperones such as proteasome maturation protein and proteasome assembly chaperone 1–2 (31) (Fig. 5 F). These are crucial for the proper assembly of the 20S standard, and immuno- and thymoproteasomes thus reflecting elevated numbers of proteasomes that were not fully assembled in PSMB10 G209R skin fibroblasts (Fig. 5 G) (3, 4). Since PSMB10 is incorporated before PSMB8 and PSMB9 (4), stalling of proteasome assembly after PSMB10 incorporation explains the lower abundance of immunopurified PSMB8 and PSMB9 but similar levels of PSMB10 (in both fully and partially assembled 20S) (Fig.5 E). Moreover, we observed an increased binding to the ubiquitin-independent proteasome activator (PA) 28αβ, PA28γ, and PA200 in G209R compared with PSMB10 WT cells (Fig. 5 H) possibly due to long-range allosteric effects, as observed previously when comparing standard and immunoproteasomes (32).

Taken together, our data strongly suggest that the PSMB10 G209R proteoform causes impaired assembly of immunoproteasomes upon IFN treatment of skin fibroblasts. Proteasomes of PSMB10 G209R skin fibroblasts have reduced proteolytic activities and bind differentially to PAs.

### Downregulation of pathways related to viral sensing and antigen presentation in IFN-treated PSMB10 G209R skin fibroblasts

**Figure 6.**
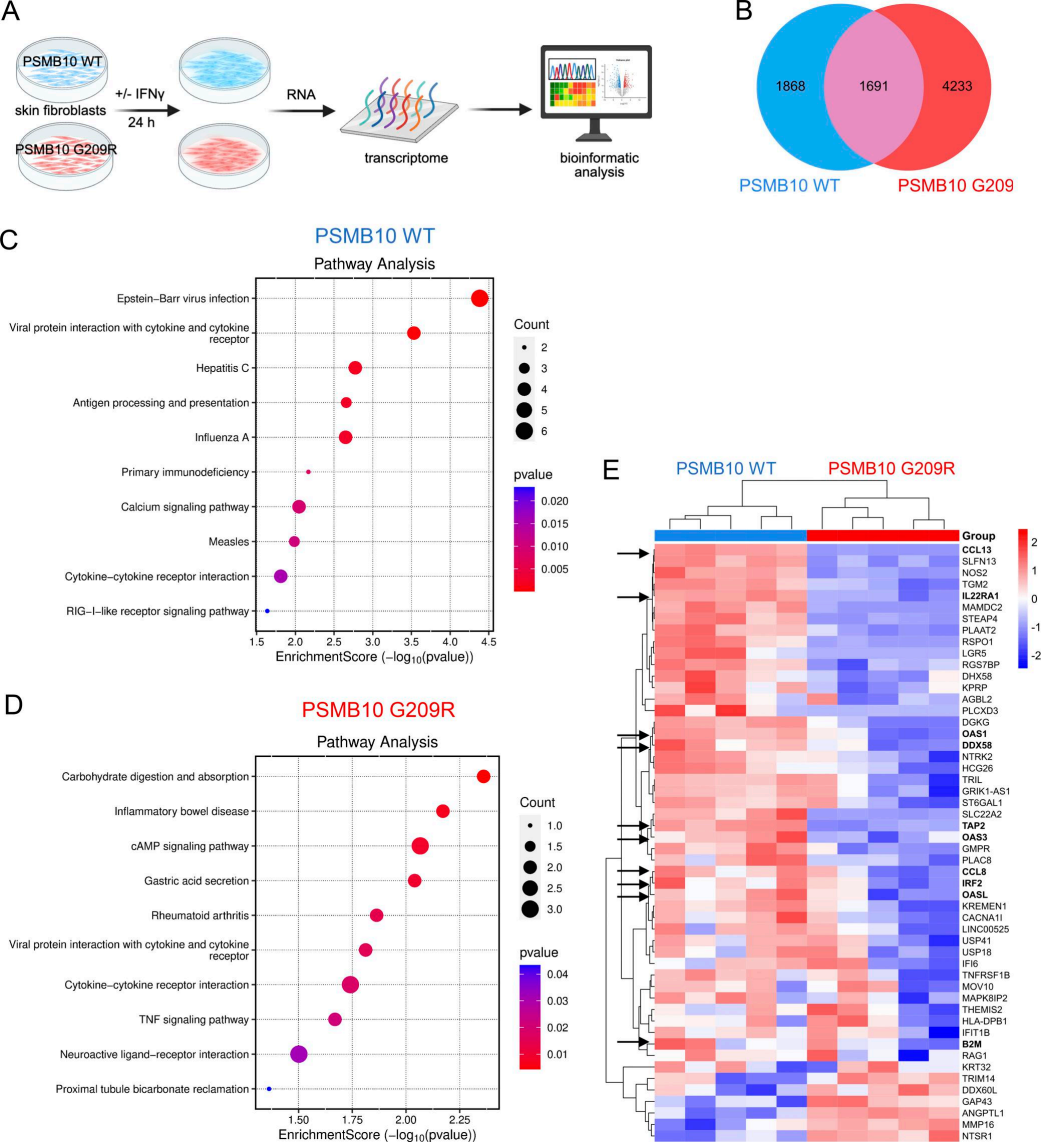
**Impaired viral sensing and antigen presentation in IFN-treated PSMB10 Gly209Arg skin fibroblasts. (A)** Comparative transcriptomic analysis of PSMB10 G209R and age-matched PSMB10 WT primary skin fibroblasts following treatment with 75 U/ml IFNγ for 24 h. Biological replicates were performed using passages 5–11 (*n* = 5, scheme generated using BioRender). Affymetrix array analysis was performed, and one-way ANOVA was used to identify significantly regulated genes (P <0.05). **(B)** Venn diagram illustrating all significantly regulated genes in PSMB10 WT and PSMB10 G209R samples upon treatment with IFNγ. **(C and D)** Pathway analysis for (C) 54 DEGs of IFNγ-stimulated PSMB10 WT and (D) 112 DEGs of IFNγ-stimulated PSMB10 G209R cells (|log2|>2, one-way ANOVA). **(E)** Heatmap of 54 genes with |log2| >2 specific to PSMB10 WT. Genes related to viral sensing and MHC class I antigen presentation are highlighted with an arrow.

We further investigated the molecular consequences of the impaired assembly and altered binding to PAs in PSMB10 G209R skin fibroblasts by transcriptome analysis. Multiple passages of PSMB10 G209R and WT skin fibroblasts were treated with IFNγ for 24 h, and the transcriptome was assessed (Fig. 6 A). We focused our analysis on differentially expressed genes (DEGs) upon IFNγ treatment to minimize the impact of the individual donors. In IFNγ-treated PSMB10 G209R skin fibroblasts, we found 4,233 DEGs as opposed to 1,868 DEGs in PSMB10 WT cells (Fig. 6 B). 1,691 DEGs that overlapped in the two transcriptomes mainly contained upregulated genes of the Janus kinase/signal transducer and activator of transcription-, interleukin (IL)-, and platelet-derived growth factor signaling pathways (data not shown).

**Figure S2.**
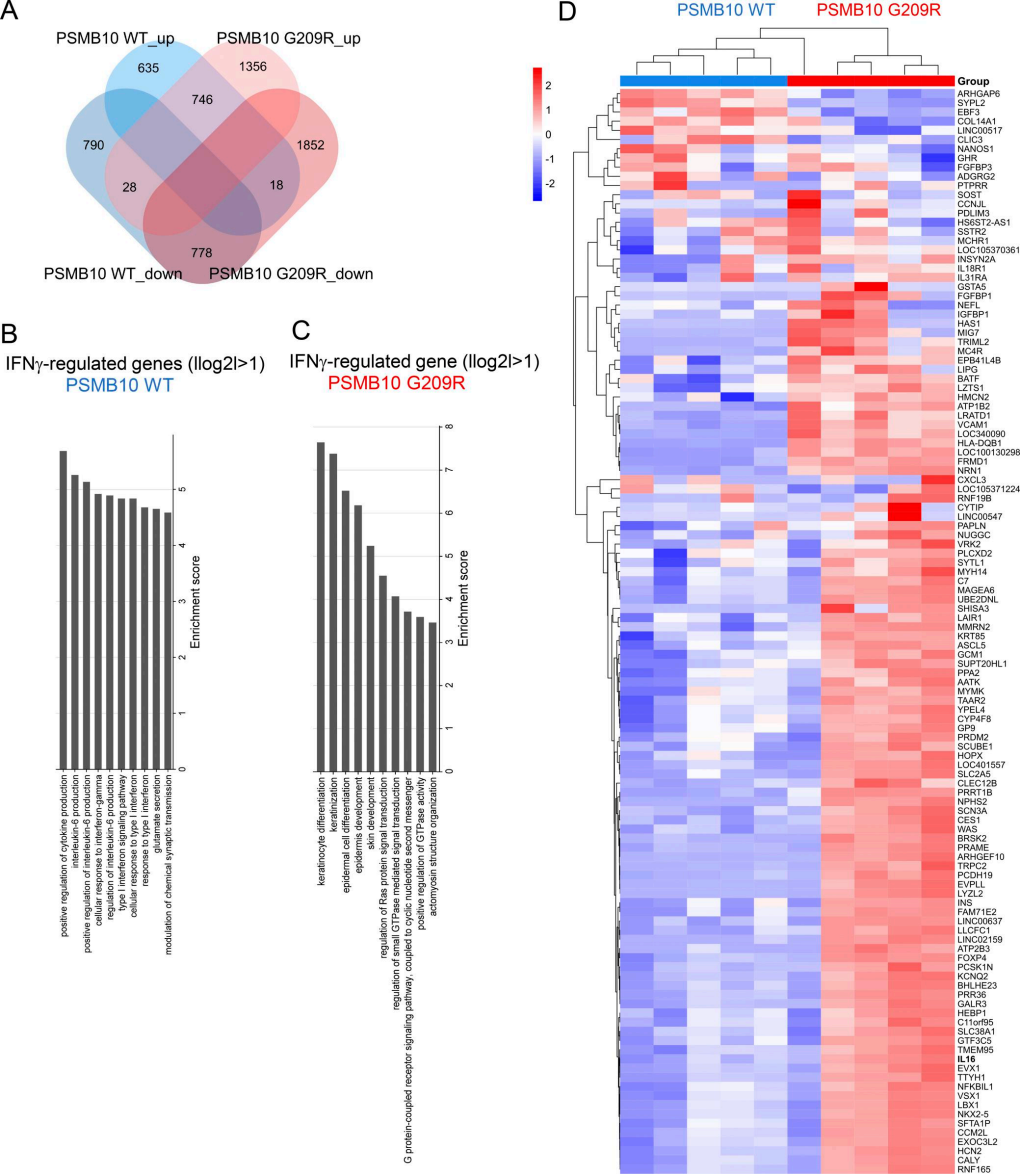
**GO and pathway enrichment analysis for IFNγ-treated PSMB10 WT and PSMB10 G209R skin fibroblasts. (A)** Venn diagram of IFNγ-induced DEGs in PSMB10 WT and PSMB10 G209R skin fibroblasts using a cutoff of |log2| >1. **(B)** GO analysis of DEGs with |log2| >1 for PSMB10 WT. **(C)** GO analysis of DEGs with |log2| >1 for PSMB10 G209R. **(D)** Heatmap of 121 genes with |log2| >2 specific to PSMB10 G209R.

To identify enriched pathways, we performed gene ontology (GO) and pathway enrichment analysis for the DEGs using a cutoff of |log2|>1 (i.e., >2× fold change compared with non–IFNγ-treated samples). With this analysis, 3,208 DEGs were found in PSMB10 G209R versus 1,425 DEGs in PSMB10 WT skin fibroblasts (Fig. S2 A). We observed strong enrichment of the proinflammatory IL-6 and IFN1 signaling pathways specific to PSMB10 WT skin fibroblasts (Fig. S2 B). In contrast, PSMB10 G209R skin fibroblasts displayed enriched regulation of epidermal skin differentiation, and G protein and KRAS signaling upon IFNγ treatment (Fig. S2, C and D).

Using a higher cutoff of |log2|>2 (i.e., >4× fold change), we identified 112 DEGs in PSMB10 G209R versus 54 DEGs in WT skin fibroblasts. Pathway analysis on these gene sets revealed strong enrichment in virus infection–related and viral sensing pathways in IFNγ-stimulated WT cells (Fig. 6 C), while IFNγ-treated PSMB10 G209R cells showed moderate enrichment for carbohydrate digestion-, receptor-, and inflammatory signaling–related genes (Fig. 6 D). A heatmap analysis of the 54 DEGs identified in PSMB10 WT cells identified C-C motif chemokine ligand 13 and nitric oxide synthase 2 (NOS2), as well as viral sensors, e.g., 2′-5′-oligoadenylate synthetase 1, and genes of the HLA-I antigen presentation pathway, e.g., antigen peptide transporter 2, to be concertedly downregulated in IFNγ-treated PSMB10 G209R compared with PSMB10 WT skin fibroblasts (Fig. 6 E).

These data indicate impaired sensing of intracellular RNA and DNA upon infection and concomitant defects in antigen presentation when cells harboring the PSMB10 G209R mutation are challenged with the proinflammatory cytokine IFNγ.

### Neonatal lymphopenia in PSMB10 G209R resolves over time and is not related to impaired thymocyte differentiation

To determine whether the patient’s T lymphopenia results from a hematopoietic stem cell (HSC)–intrinsic or HSC-extrinsic defect in T cell development, we took advantage of artificial thymic organoid (ATO) assays that support ex vivo differentiation of CD34^+^ HSCs into TCR-expressing cells. This ATO assay has been successfully employed to determine whether patients with selective T lymphopenia of unknown genetic origin have a hematopoietic or a thymic stromal/organogenesis disorder (33, 34).

**Figure 7.**
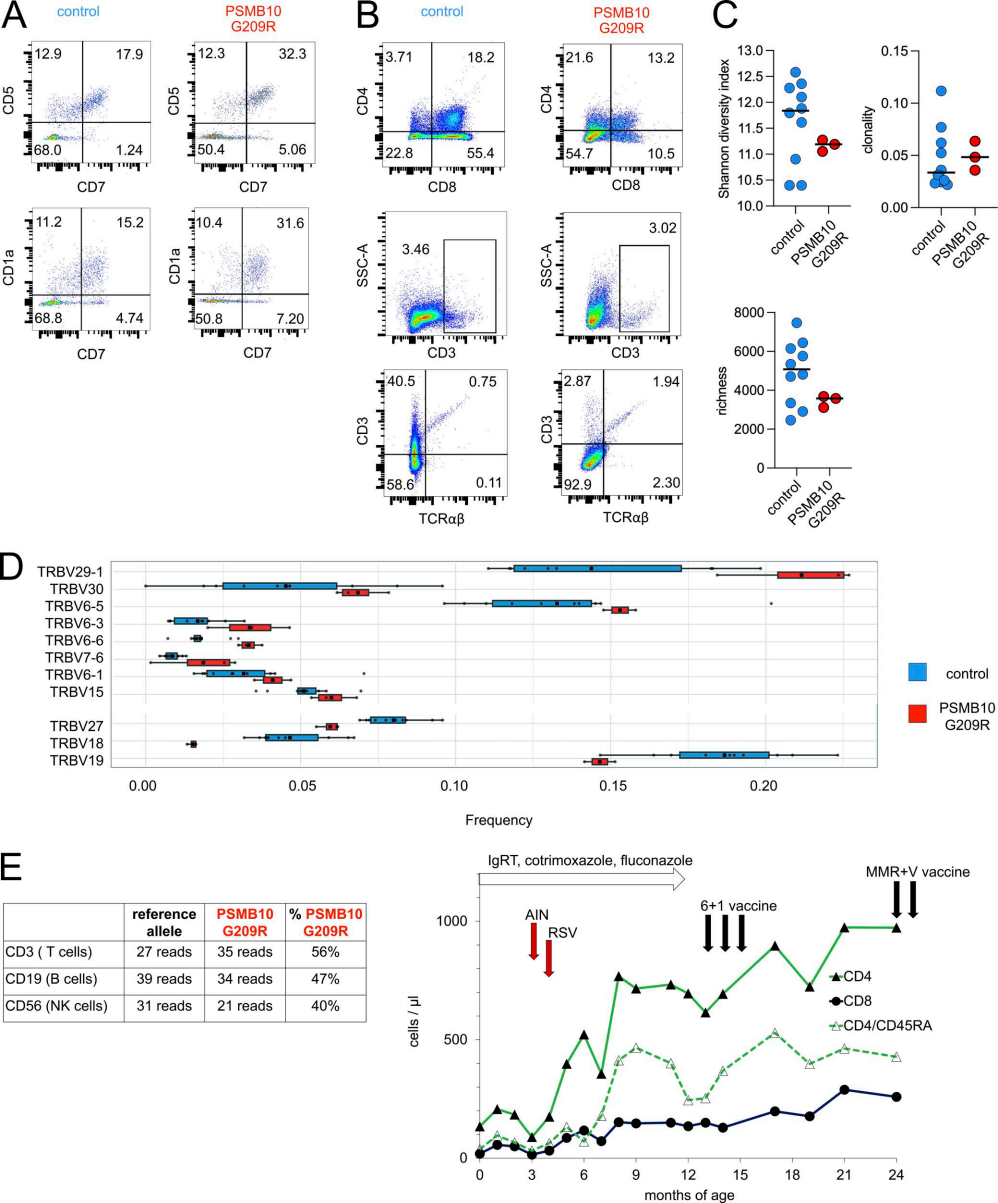
**Neonatal lymphopenia in PSMB10 G209R resolves over time and is not related to impaired thymocyte differentiation. (A and B)** Flow plots showing ex vivo differentiated proportions of patient- and healthy donor–derived cells (gated within live CD45^+^ cells) expressing (A) early T cell progenitor differentiation markers CD5, CD7, and CD1a, and (B) late T cell differentiation markers CD4, CD8, CD3, and TCRab from PSMB10 WT and PSMB10 G209R HSCs in ATOs. **(C)** Bulk TCR repertoire sequencing of PSMB10 G209R compared with 10 age-matched individuals shows similar clonality, richness, and diversity. **(D)** VJ architecture of TCR repertoire displays mild skewing as compared to the control samples. This skewing is mainly mediated by differential usage of distinct T cell receptor beta variable region (TRBV) (enrichment of TRBV29-1, TRBV30, TRBV6-5; reduced usage of TRBV27, TRBV18, TRBV19) (see also Fig. S5 D). **(E)** T cell reconstitution and clinical course of the PSMB10 G209R patient in the first 24 mo of life. Total CD8, total CD4, and naïve CD4 cell counts, clinical events (AIN = autoimmune neutropenia, RSV = respiratory syncytial virus infection), and medical interventions (white flash in the top: prophylaxis given; black arrows = vaccines given 6 + 1 = inactivated vaccine again tetanus, diphtheria, pertussis, poliomyelitis, Haemophilus influenzae type B, hepatitis B, and *S. pneumoniae*; MMR+V = live vaccine against measles, mumps, rubella, and chickenpox) are shown.

**Figure S3.**
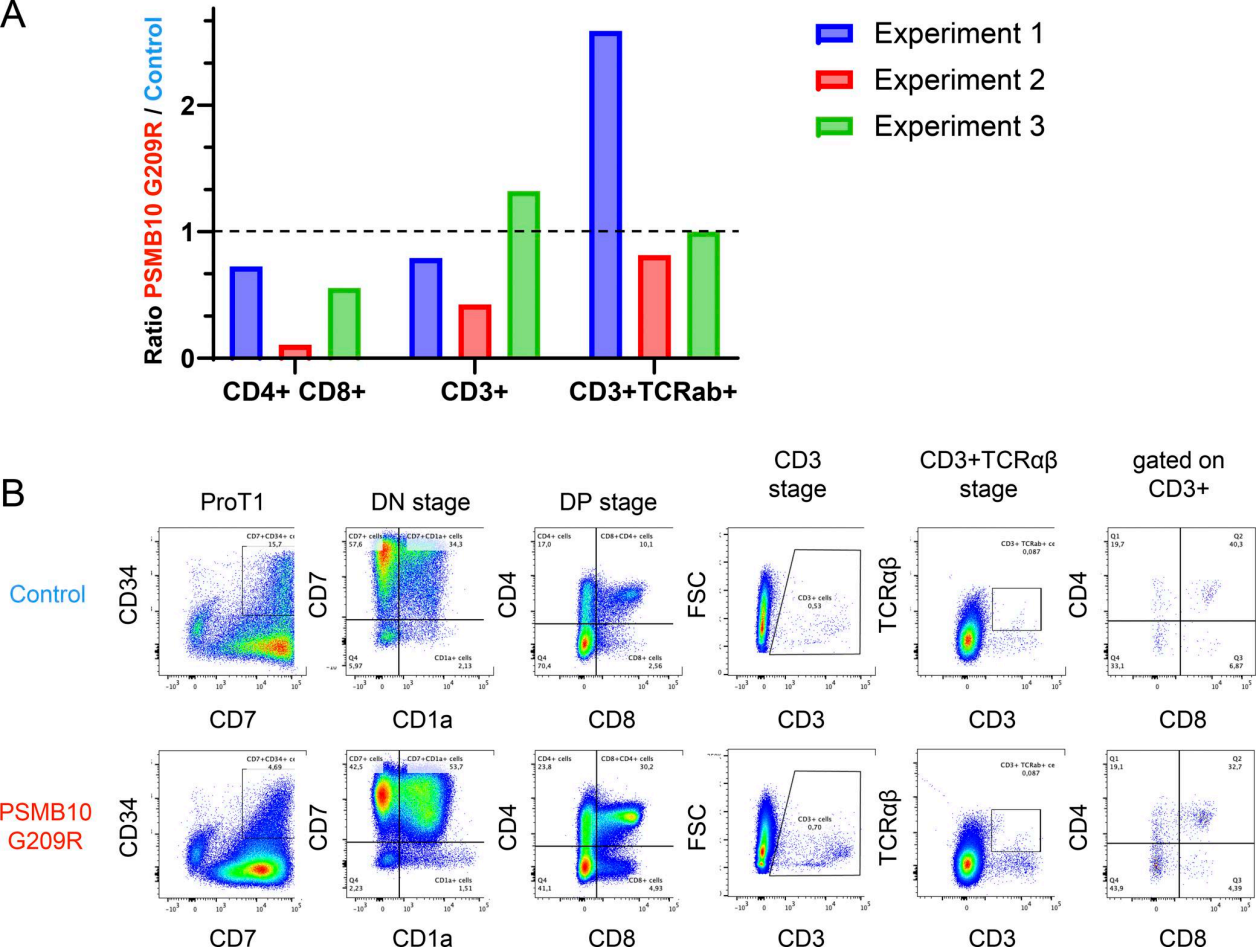
**Ex vivo differentiation of CD3^+^TCR^+^ cells from PSMB10 WT and PSMB10 G209R HSCs in ATOs. (A)** Histograms showing the ratios of DP CD4^+^CD8^+^ cells, CD3^+^ cells, and CD3^+^TCRαβ^+^ cells among live CD45^+^ cells in cocultures of PSMB10 G209R versus PSMB10 WT, based on three independent experiments (see also Fig. 7, A and B). The dashed line indicates a ratio of 1. **(B)** Flow cytometry pseudocolor plots displaying the proportions of differentiating cells (early T cell progenitor markers CD5, CD7, CD1a, and late T cell differentiation markers CD4, CD8, CD3, TCRαβ) from patient- and healthy donor–derived control cells (gated on live CD45^+^ cells) in an ATO model performed in a second laboratory. Consistent with results shown in Fig. 7, A and B, there is no differentiation arrest, and mature CD3^+^TCR^+^ T cells develop from both PSMB10 WT and PSMB10 G209R HSCs.

Coculture of primary PSMB10 G209R and PSMB10 WT HSCs with human Notch ligand–expressing stromal cell lines resulted in successful ex vivo T cell differentiation with all developmental stages present among the differentiating cells, i.e., CD5^+^CD7^+^ and CD1a^+^CD7^+^ lymphoid progenitors, CD4^+^CD8^+^ double-positive cells, and CD3^+^CD4^+^ and CD3^+^CD8^+^ TCR-expressing single-positive cells (Fig. 7, A and B; and Fig. S3, A and B), suggesting that the patient’s T lymphopenia may be secondary to a thymic stromal cell defect (35).

**Figure S4.**
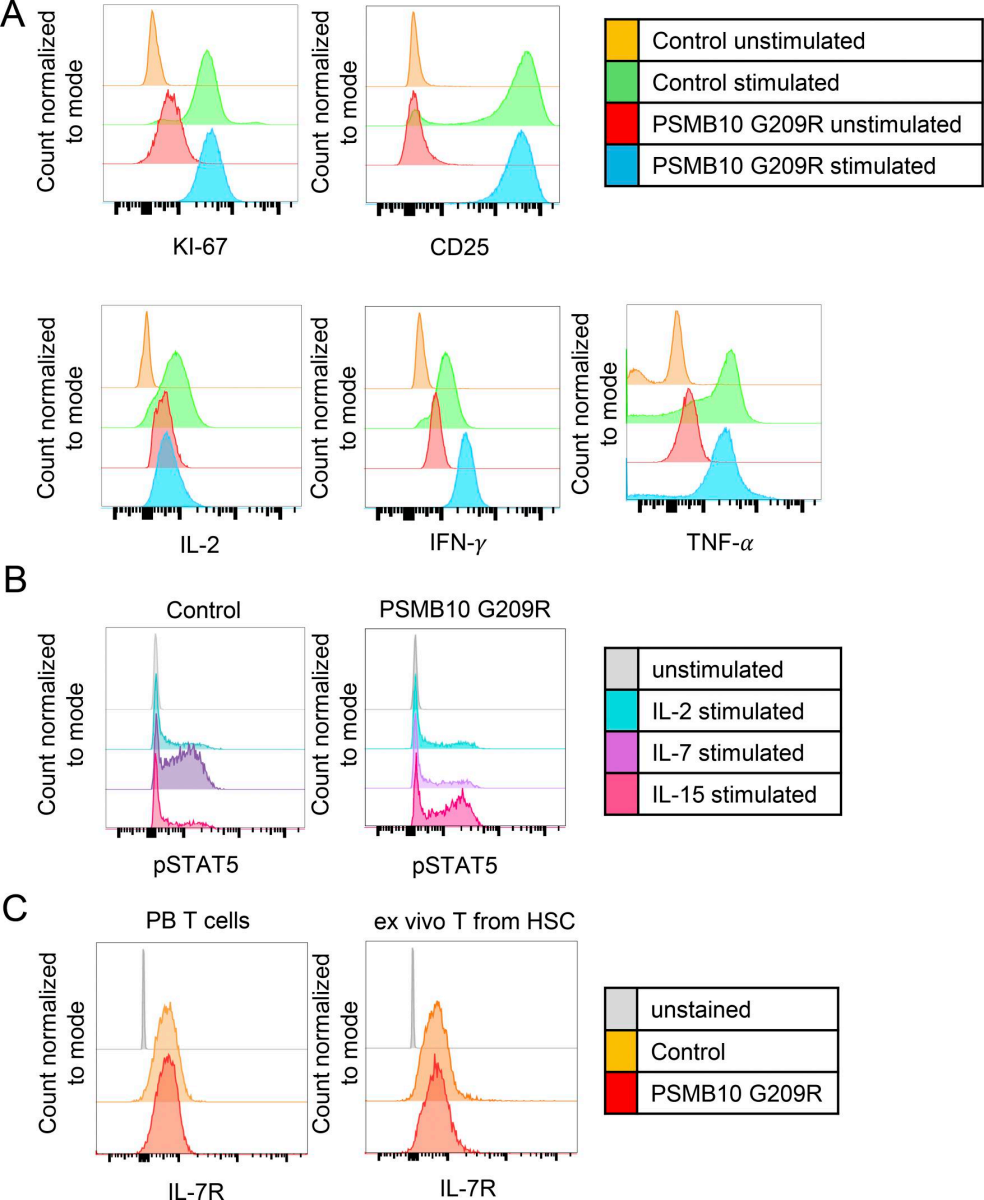
**PSMB10 G209R T cells show normal immune phenotype and function. (A)** Histograms displaying the expression of Ki-67, CD25, IL-2, IFNγ, and TNF in both PSMB10 G209R and PSMB10 WT peripheral blood before and after stimulation with CD3/CD28 beads, gated on CD4^+^ and CD8^+^ SP cells. **(B)** Phospho-STAT5 upregulation in CD3^+^ cells in response to IL-2, IL-7, and IL-15 stimulation in both PSMB10 G209R and PSMB10 WT peripheral blood. **(C)** Expression of IL-7R (CD127) in PSMB10 G209R T cells compared with PSMB10 WT T cells, and in ex vivo generated T cells from PSMB10 G209R HSCs compared with ex vivo generated T cells from PSMB10 WT HSCs.

Over a 12-mo time course, the PSMB10 G209R patient became T cell–proficient with normalization of T cell counts and TREC levels (Table 1). At the age of 51 mo, we observed normal T cell responses with CD25 upregulation, increased Ki-67 expression, and cytokine production (IL-2, IFNγ, and TNF) after TCR stimulation with anti-CD3/CD28-coated beads (Fig. S4 A), demonstrating that PSMB10 G209R patient T cells are capable of activation and proliferation. Additionally, as abnormal IL-7 signaling with reduced IL-7 receptor (IL-7R) expression has been described in one patient with a heterozygous PSMB10 G201R variant and OS (8), we also measured phosphorylation of STAT5 (pSTAT5) after stimulation with IL-7, IL-2, and IL-15. We observed similar pSTAT5 expression with all 3 stimuli in PSMB10 G209R and PSMB10 WT T cells (Fig S4 B). Baseline IL-7R expression was not altered on PSMB10 G209R compared with PSMB10 WT T cells that had been differentiated from peripheral blood ex vivo (Fig. S4 C).

**Figure S5.**
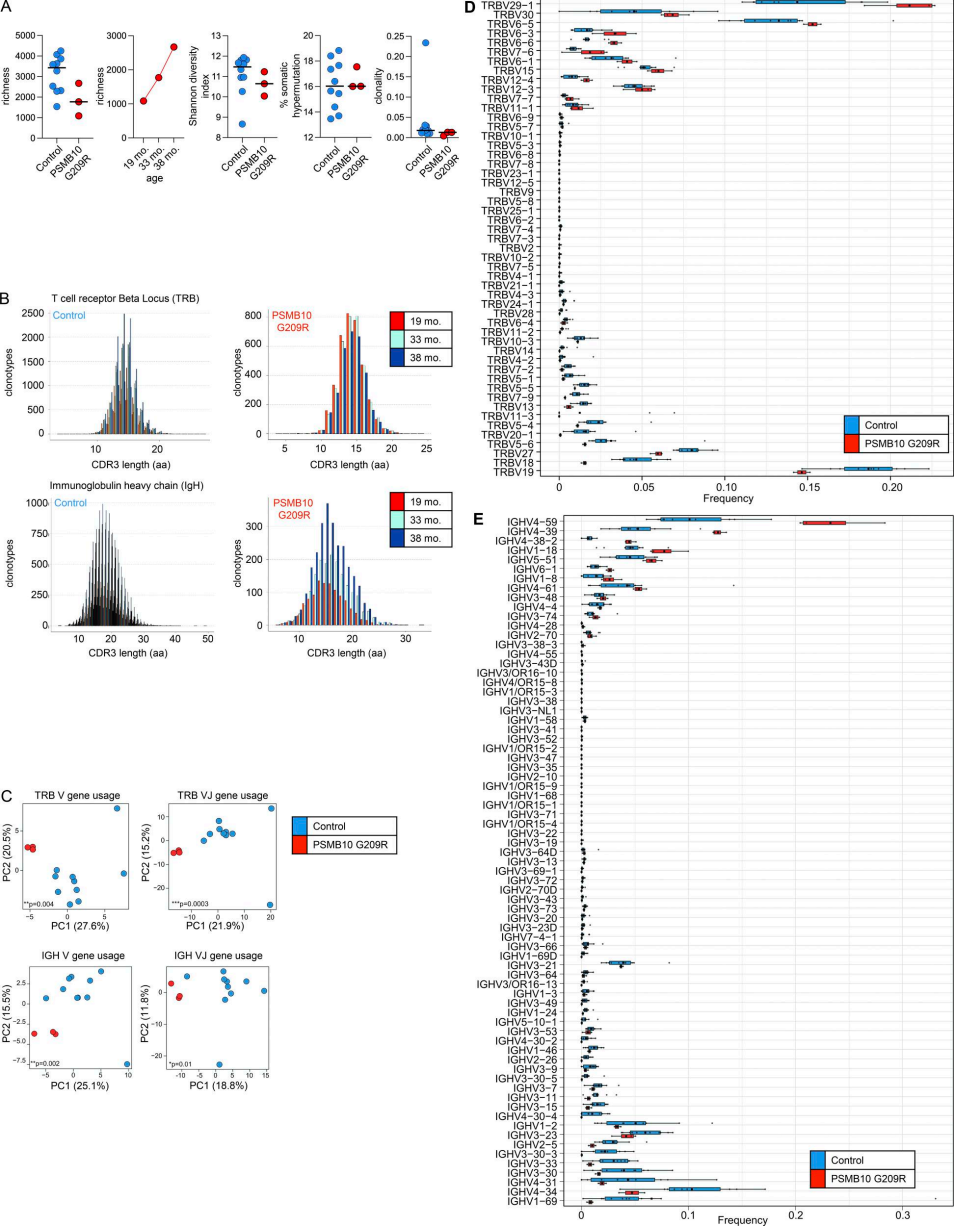
**Immune repertoire metrics from bulk TCR and BCR sequencing. (A)** BCR repertoire analysis of the PSMB10 G209R patient compared with age-matched controls (*n* = 10) shows similar overall metrics, including richness, diversity, somatic hypermutation, and clonality. **(B)** CDR3 length distributions in the TRB and IgH loci across three time points in the PSMB10 G209R patient compared with controls. **(C)** PCA of BCR and TCR V and VJ gene usage architecture. Statistics: Pillai–Bartlett trace from MANOVA across all principal components. **(D and E)** Median frequencies of TRBV and IGHV gene usage in the PSMB10 G209R patient compared with controls. The VJ gene usage in the TCR repertoire shows mild skewing relative to control samples. PCA, principal component analysis; MANOVA, multivariate analysis of variance.

Bulk immune repertoire TCR and B cell antigen receptor (BCR) sequencing at 19, 33, and 38 mo of age as compared to 10 age-matched individuals showed normal global repertoire metrics such as diversity (Shannon), clonality, richness, and somatic hypermutation (TCR; Fig. 7 C, BCR; Fig. S5 A). The distribution of the complementary-determining region 3 (CDR3) lengths was normal (Fig. S5 B) (36). The richness of the BCR repertoire was within the lower range at the first sampling time point but steadily normalized over time (Fig. S5 A). However, the VJ architecture of the TCR and BCR repertoires displayed a skewing as compared to the control samples (Fig. 7 D and Fig. S5 C). This skewing was mainly mediated by differential usage of distinct TRBV (enrichment of TRBV29-1, TRBV30, TRBV6-5; reduced usage of TRBV27, TRBV18, TRBV19) and immunoblobulin heavy chain variable region (IGHV) (enrichment of IGHV4-59, IGHV4-39, IGHV4-38-2; reduced usage of IGHV4-31, IGHV4-34, IGHV1-69) rearrangements (Fig. 7 D; and Fig. S5, D and E). Independently of bulk repertoire sequencing, the TCR repertoire was repeatedly assessed from early infancy onward by flow cytometry–based analyses at 1 (Fig. 1 C) and 2 wk of age and subsequently at 5, 15, 30, 33, 38, and 46 mo of age. Across all time points, these analyses consistently demonstrated a polyclonal TCR repertoire without evidence of an oligoclonal expansion (not shown).

To investigate somatic reversions that might have allowed for T cell reconstitution over time, we sorted T, B, and NK cells, isolated genomic DNA, and performed whole-exome sequencing. Both PSMB10 G209R and WT sequences were found in all samples, and their distribution was in line with unaltered persistence of the monoallelic missense variant in *PSMB10* c.625G>A (Fig. 7 E).

In the clinical course with a follow-up of 48 mo, the patient developed autoimmune neutropenia at the age of 3 mo and a physiological infection with respiratory syncytial virus at 4 mo of age that both resolved spontaneously. Homely isolation, prophylactic cotrimoxazole, fluconazole, and Ig replacement therapy were discontinued at 12 mo of age (Fig. 7 F).

Protein-based dead vaccines against tetanus, diphtheria, pertussis, poliomyelitis, Haemophilus influenzae type B, and hepatitis B, as well as protein-conjugated polysaccharide dead vaccines against *Streptococcus pneumoniae*, were given at the age of 12, 13, and 14 mo, and persistent seroconversion was documented. Next, attenuated viral live vaccines against measles, mumps, rubella, and chickenpox were given at the age of 24 mo without adverse events and with protective seroconversion (Table 1 and Fig. 7 F).

Overall, these data are in line with a lymphocyte-extrinsic cause of neonatal lymphopenia most probably attributable to an immunoproteasome defect in thymic epithelial cells, which leads to a delayed constitution of a proper T cell system. At the latest follow-up at the age of 66 mo, the patient showed no signs of pathological infection susceptibility, autoinflammation, or autoimmunity.

## Discussion

TREC-based newborn screening in combination with NGS has substantially improved the diagnostic rate and treatment outcome of SCID in recent decades (13, 37). However, it has become increasingly clear that TREC-based newborn screening also identifies non-SCID neonatal T lymphopenia. The latter can be caused by less severe congenital hemato/lymphopoietic or thymic stromal cell and/or organogenesis defects (14, 38), or can be secondary to an array of underlying syndromic disorders or biochemical exposures (13, 14, 39, 40). It is therefore mandatory to establish a precise diagnosis in a newborn with severe T lymphopenia to choose the most suitable treatment strategy be it either allogeneic hematopoietic cell transplantation (HCT), allogeneic thymus transplantation, or conservative treatment (14, 41). In addition, the combination of TREC-based newborn screening with NGS and thorough biomedical research is a unique tool to advance our knowledge on human immunity (42).

We here report a case of transient neonatal T lymphopenia detected by TREC-based newborn screening that is associated with a monoallelic de novo PSMB10 G209R private variant. The substitution of the small glycine residue at position 209 of PSMB10 into a bulky and positively charged arginine occurred at a highly conserved position and surface surrounding of the protein. In silico modeling and MDS of this substitution predicted impaired assembly of the immunoproteasome which we confirmed by computational docking, MS, and biochemical analysis in skin fibroblasts where immunoproteasome assembly was induced by IFN treatment.

Human PSMB10 variants have been associated with distinct clinical entities depending on their mode of inheritance and molecular consequence. Four children presenting with PRAAS were reported with biallelic loss-of-function (LOF) PSMB10 variants encoded by five distinct alleles (P14S, P14del, C83Lfs*123, G187D, and c.710+1G>C) that were not expressed, processed, or incorporated into the mature immunoproteasome. They largely responded to Janus kinase inhibition and displayed no signs of cellular immunodeficiency. Parents with monoallelic PSMB10 LOF variants were healthy (9, 43). The authors concluded that constitutively expressed PSMB7 partially compensated for PSMB10 LOF alleles that upon IFN induction physiologically endow immunoproteasomes with increased proteolytic capacity in nonhematopoietic tissues. In the setting of biallelic PSMB10 LOF alleles, this compensation was thought to be insufficient to maintain proteohomeostasis leading to proteotoxic stress underlying PRAAS.

Furthermore, six infants with T^−^B^−^NK^+/−^ SCID and OS-like disorder were reported and all of them had de novo monoallelic PSMB10 variants encoded by two distinct alleles (D56H and G201R). Upon in silico modeling and basic biochemical analysis, these alleles were shown to be expressed and discussed to impair immunoproteasome β-ring pairing (8, 10). PSMB10 G201R ex vivo CD34^+^ peripheral blood cells were efficiently differentiated in an ATO system (*n* = 1) into early developmental stages indicating a potential extra-hematopoietic etiology of lymphocytopenia; however, late developmental stages were slightly reduced suggesting an additional hematopoietic-intrinsic defect. All patients had impaired TCR and BCR repertoire diversity, T lymphocyte clonality, eosinophilia, opportunistic infections, and build-up dermal, gastrointestinal, and systemic inflammation. Six patients were treated with allogeneic HCT and with the exception of one patient, that before HCT had revertant mosaicism overlapping the PSMB10 locus in 15–20% of blood cells, four succumbed to peri- and posttransplant inflammatory complications of the skin and gastrointestinal tract including graft versus host disease. One developed debilitating inflammatory neurological damage on short-term follow-up. The authors concluded that DN PSMB10 variants were incorporated into mature immuno- and thymoproteasomes and thereby interfered with compensatory substitution by PSMB7. They argued that the clinical posttransplantation phenotype of the patients pointed toward an extra-hematopoietic immunoproteasome dysfunction impacting on proteohomeostasis, especially in inflamed epithelial cells akin to PRAAS, and that in analogy to the Psmb10-deficient mice, thymic cortical epithelial cells might be dysfunctional or missing (25).

Very recently, Fournier et al. reported three additional patients with de novo monoallelic PSMB10 variants (D205A and S208F) who developed profound immunodeficiency with prominent extra-hematopoietic manifestations, including severe endothelial and hepatic disease, and similarly high mortality following HCT underscoring the phenotypic variability associated with monoallelic PSMB10 variants (11).

In contrast to previously reported patients with monoallelic PSMB10 variants, our patient exhibited transient neonatal T lymphopenia with spontaneous normalization of thymic output, naïve T cell counts, and TCR repertoire diversity.

It is known that somatic revertant mosaicism in hematopoietic cells can mitigate clinical phenotypes of IEI (44). Somatic reversion in epithelial cells, e.g., of the skin, leads to clinical mosaicism with corrected skin spots on the background of diseased skin but not to correction of the entire epithelial surface (45). In the patient reported here, we excluded somatic revertant mosaicism in hematopoietic cells; however, low-level somatic reversion within thymic or other nonhematopoietic compartments cannot be formally excluded and may represent one possible explanation for the favorable clinical course.

While impaired assembly of the immunoproteasome has been suggested also for some other PSMB10 variants (8, 9, 10, 11), our combined computational, biochemical, and biophysical data provide strong evidence consistent with a DN effect of PSMB10 G209R on immunoproteasome assembly and function. Accordingly, immunoproteasome and thymoproteasome assembly occurs in a stepwise fashion and is assisted by several assembly chaperones. Assembly starts with the formation of the 20S α-ring of the proteasome to which the inactive precursor forms of PSMB10, PSMB9, and subsequently PSMB8 (immunoproteasome) or PSMB11 (thymoproteasome) bind together with the remaining noncatalytic β subunits (4). Two half-proteasomes then assemble in a mirrored orientation to form a mature and active proteasome after autocatalytic removal of the precursor sequences (4). In this assembly process, PSMB10 plays a crucial role in facilitating assembly of both the immuno- and thymoproteasome. In the absence of PSMB10, the constitutive counterpart PSMB7 can be integrated into an intermediate type of immunoproteasome (46). Our TD-MS analysis of immunopurified proteasome complexes revealed that cells were able to incorporate PSMB10 G209R into the immunoproteasome but only to a minor extent of 77:23 instead of the expected 50:50 incorporation of PSMB10 WT and G209R alleles (29). We also observed reduced incorporation of subsequent PSMB9 and PSMB8 subunits and accumulation of assembly intermediates containing assembly chaperones, which substantiates the DN effect of PSMB10 G209R on immuno- and thymoproteasome assembly.

Our data are fully in line with the TUB6 mouse model that harbors a Psmb10 G170W substitution that is homologous to the human PSMB10 G209R substitution reported here. Psmb10 G170W also results in stalled immuno- and thymoproteasome assembly (25), and TUB6 mice display athymia with systemic autoinflammation and SCID in the biallelic state and hypothymia with selective T lymphopenia in the monoallelic state (25). In contrast, mice with a biallelic knockout of Psmb10 neither show a thymic defect nor T lymphopenia. In these mice, Psmb10 is replaced by the standard subunit Psmb7 thereby maintaining the functional integrity of the thymus and thus arguing against Psmb10 haplo- or diploinsufficiency at least in terms of quantitative thymopoiesis (47). We thus suggest that the observed neonatal T lymphopenia is consistent with altered immune- and thymoproteasome function resulting from impaired assembly.

We further demonstrate that monoallelic DN PSMB10 G209R skin fibroblasts formed immunoproteasomes upon IFN induction that had reduced activities and bound differentially to PAs, suggesting that the resulting complexes had an altered substrate specificity and proteolytic activity. Due to the low expression of the immunoproteasome in skin fibroblasts, we did not observe major transcriptomic changes at baseline. However, we cannot rule out that higher baseline expression of the immune- or the thymoproteasome in DN PSMB10 G209R immune or thymic cells causes a more pronounced transcriptomic deregulation. Other PSMB10 and PRAAS mutations exhibit activation of IFN1 signaling, a hallmark of these types of interferonopathies, which was, however, missing in our patient clinically and in peripheral blood samples (10, 48, 49). In contrast, IFN-mediated induction of the immunoproteasome in DN PSMB10 G209R skin fibroblasts significantly altered the transcriptome as compared to WT cells. We observed impaired induction of multiple genes involved in antiviral immunity such as viral sensors, and HLA class I antigen presentation– and cytokine-related genes in DN PSMB10 G209R cells. These findings suggest that the reduced immunoproteasome assembly and activity caused by the DN PSMB10 G209R variant may contribute to an impaired development of cTECs and mTECs and interfere with CD8 T cell–mediated response to viral infection (50).

This might especially compromise immunity against CMV and EBV infection that critically depends on immunoproteasome-mediated HLA class I antigen presentation and CD8 T cell expansion (51, 52, 53). Notably, the patient reported here remains CMV-negative so far and has not displayed hemophagocytic lymphohistiocytosis (HLH)-like episodes. It is noteworthy that NOS2, which at present is the only known monogenic IEI with selective and nonredundant predisposition toward severe CMV infection, is downregulated in IFN-treated DN PSMB10 G209R skin fibroblasts (54). Furthermore, upon IFN-mediated expression in nonhematopoietic cells, the DN PSMB10 G209R might lead to an elevated susceptibility to infection-mediated hyperinflammatory immune dysregulation. This is reflected by a gene dosage continuum observed in the murine models discussed above (25, 47) and might as well have contributed to the hyperinflammatory OS phenotype (55) observed in humans with monoallelic PSMB10 variants (8, 10, 11).

TCRs recognize antigenic peptides presented by HLA complexes, and the naïve human TCR repertoire has been estimated to encompass ∼10^8^ clonotypes (56). The importance of TCR diversity has been illustrated by the immune response toward both highly endemic and emerging infections such as CMV and severe acute respiratory syndrome coronavirus 2, respectively, allowing for the selection of best-fit clonotypes to control these pathogens and to establish long-term memory (57, 58, 59). In the context of IEIs caused by recombination-activating gene 1/2 (RAG1/2) deficiency, the degree of repertoire diversity restriction, skewed usage of variable (V), diversity (D), and joining (J) gene segments, and altered CDR3 length distribution dictate the phenotypic spectrum ranging from SCID over OS and CID to autoimmunity only (60). In the setting of allogeneic HCT for SCID, poor TCR diversity after transplantation is predictive of insufficient immune reconstitution (61). In addition to resolution of mere neonatal T lymphopenia, we showed normal global repertoire metrics such as diversity (Shannon), clonality, richness, and CDR3 length distribution for the DN PSMB10 G209R patient consistent with a robust and long-lasting immune reconstitution. However, the VJ architecture of the TCR and BCR repertoires displayed a skewing, and even though this may be overestimated because the three DN PSMB10 G209R samples were from one individual only, long-term follow-up of the patient will be important to assess potential risks of autoimmunity or malignancy, as described in other settings of altered TCR architecture (15, 62). An additional possibility is that the dysfunctional immunoproteasome generates an altered pool of HLA class I peptides under inflammatory conditions, thereby reshaping TCR repertoire architecture without necessarily impairing antigen-specific responses. Similar observations have been reported in murine models (63, 64).

We conclude that monoallelic DN PSMB10 G209R variant is associated with impaired immuno- and thymoproteasome assembly and supports a model in which altered proteasome function within the thymic stromal microenvironment, potentially inside the thymic epithelial cells, contributes to neonatal T lymphopenia. Without newborn screening, the patient might not have come to clinical attention in the absence of severe infectious or inflammatory manifestations. The longitudinal follow-up suggests that in rare instances, profound neonatal T lymphopenia associated with monoallelic DN PSMB10 variants may represent a dynamic and potentially reversible condition. As illustrated by the patient presented here, minimizing infectious and inflammatory triggers during early infancy may be particularly important, given the IFN-inducible nature of PSMB10 and the potential impact on nonhematopoietic compartments. Preventive strategies such as Ig replacement, anti-infective prophylaxis, avoidance of live vaccines, and careful monitoring may therefore be reasonable in selected cases. In patients with overt inflammatory phenotypes, targeted immunomodulatory approaches could be considered on an individual basis. In contrast, conditioning-based alloHCT may not fully address extra-hematopoietic disease components and warrants careful evaluation (10, 11). Whether thymus transplantation represents a viable option in cases of persistent thymic dysfunction remains to be determined.

In summary, we demonstrate that the monoallelic PSMB10 G209R variant impairs immunoproteasome assembly in a manner consistent with a DN effect and is associated with transcriptomic changes indicative of impaired sensing of intracellular RNA and DNA and concomitant defects in antigen presentation. Nevertheless, affected HSCs retain their ability to differentiate ex vivo into mature TCR-expressing lymphocytes, giving rise in vivo to functional T cells with a polyclonal, yet slightly altered, TCR repertoire. Together, these observations expand the phenotypic spectrum of monoallelic PSMB10 variants and highlight the dynamic nature of early-life thymic function.

## Materials and methods

### TREC quantification and genetics

TREC-based newborn screening was performed as part of the prospective newborn screening for SCID and T lymphopenia in Germany (13). TRECs were again assessed at 51 mo on fluorescence-activated cell-sorted CD3 cells from PBMCs (65).

Exome sequencing was performed at the Dr. von Hauner Children’s Hospital next-generation sequencing platform. Briefly, genomic DNA was isolated from whole blood samples or magnetic cell separation (MACS)-purified CD3, CD19, and CD56 cells with the iPrep PureLink gDNA Blood kit (Thermo Fisher Scientific). Preparation of whole-exome libraries was done using the SureSelect XT Human All Exon V6+UTR kit (Agilent Technologies), and they were subsequently sequenced with a NextSeq 500 platform (Illumina) to an average coverage depth of 90×. Bioinformatics analysis was done using the Burrows–Wheeler Aligner (version 0.7.15), Genome Analysis ToolKit (version 3.6), and Variant Effect Predictor (version 89). For allele frequency filtering, public (e.g., GnomAD) and in-house (∼5,600 exomes) databases were used. *PSMB10* sequence variants were validated by Sanger sequencing (Applied Biosystems SeqStudio Flex Genetic Analyzer) using the following primers: forward 5′-3′: 5′-TTA​ACA​GCA​GAG​AGA​GCC​CG-3′ and reverse 5′-3′: 5′-TCC​AGC​TCT​CAC​CTC​TTC​AC-3′. UniPro UGENE was used to visualize DNA sequences.

### Immunophenotyping

Whole blood was taken from the patient and anticoagulated with EDTA and processed within 24 h. Immunophenotyping of lymphocyte subsets was performed by incubating the samples with fluorochrome-conjugated monoclonal antibodies targeting surface markers of lymphocyte subsets after red blood cell lysis. Beckman Coulter conjugates were: anti-CD3-FITC and APC-A750 (UCHT1), anti-CD16-PE (3G8), anti-CD56-PE (N901), anti-TCR-PAN γ/δ-PC5.5 (Immu510), anti-TCR-PAN α/β-APC (IP26A), anti-CD4-PB (13B8.2), anti-CD8-KRO (B9.11), anti-CD20-APC-A700 and PC7 (B9E9), and anti-CD45-KRO (J33). TCR Vβ staining was performed using a panel of antibodies (Beta Mark TCR Vbeta Repertoire, Beckman Coulter). Flow cytometry–based TCR Vβ repertoire analysis was performed at 1 and 2 wk and at 5, 15, 30, 33, 38, and 46 mo of age. Additionally, anti-CD25 (2A3), anti-Ki-67 (Ki-67), anti-IFNγ (4S.B3), anti-TNF (Mab11), anti-IL-2 (MQ1-17H2), anti-IL-7Ra (A019D5), and anti-phospho-STAT5 (Tyr694) were used to investigate T cell functionality with staining of intracellular markers performed using Cytofix/Cytoperm Fixation/Permeabilization Kit (BD). Data acquisition was performed using a Navios EX (Beckman Coulter) or a FACSymphony(BD) flow cytometer. Cell staining and flow cytometry–based analysis of T cells or PBMCs cultured in complete medium in the presence of PHA or anti-CD3/CD28-coated beads were performed as described earlier (66).

### Bulk immune repertoire sequencing

Immune repertoire sequencing was performed using 250–500 ng genomic DNA isolated from PBMCs. For amplification of rearranged Ig heavy chain (IGH) and T cell receptor β (TRB) loci, the BIOMED2-FR1 (IGH) and TRB-B were applied. PCR was performed using Phusion High-Fidelity DNA Polymerase (Thermo Fisher Scientific). After gel electrophoretic separation and purification, PCR amplicons were subjected to a second barcoding PCR for the addition of 7-nucleotide single indices and Illumina adapter sequences. Amplicons were pooled at 4 nM and quality-assessed on 2100 Bioanalyzer (Agilent Technologies). Sequencing was performed on an Illumina MiSeq (paired end, 2 × 301 cycles, v3 chemistry). Rearranged IGH and TRB loci were aligned using the MiXCR framework with the IMGT library v3 as a reference for IGH and the default MiXCR library as a reference for TRB loci. A clonotype was defined as a unique CDR3 nucleotide sequence. Sequences with <2 read counts or nonproductive reads were not included in downstream analyses. For analyses, reads were proportionally normalized to 20,000 (IGH) or 50,000 (TRB) counts. Immune repertoire metrics were calculated as described previously (58, 67). V gene segments exhibiting <98% identity to the germline sequence were considered hypermutated. All analyses were performed using R 4.3.1 and RStudio (version 1.1.456), and the tcR, ade4, tidyverse, and immunarch packages. Sequencing was performed with peripheral blood at 19, 33, and 38 mo of age. As an age-matched reference population, 10 individuals from the LoewenKIDS birth cohort (median age 11 mo, range 11–48; 6 female, 4 male) were used (68).

### Ex vivo T lymphopoiesis assays

Ex vivo T lymphopoiesis was assessed following coculture of CD34 HSCs with MS5 or OP9 murine stromal cell lines, expressing human Notch ligands (respectively hDLL1 or hDLL4) in ATO as previously described (33, 34). Briefly, healthy donor control and patient CD34 HSCs were purified from fresh or frozen peripheral blood samples using a MACS kit or by fluorescence-activated cell sorting (BD FACSAria Cell Sorter). CD34 HSCs were cocultured with the above-mentioned stromal cells for a period of 5–6 wk in cytokine-supplemented media. Differentiating cells were then harvested from the insert for fluorescence-activated cell sorter analysis (BD FACSymphony flow cytometer) of cell surface markers expressed at subsequent stages of T cell development: anti-human CD34 (APC-Cy7, mouse, BioLegend), anti-human CD3 (BV421, mouse, BD Horizon), anti-human CD45 (BV510, mouse, BD Horizon), anti-human CD1a (APC, mouse, BD Pharmingen), anti-human CD5 (PE, mouse, Beckman Coulter), anti-human CD7 (BV450, mouse, BD Horizon), anti-human TCRγδ (PE, mouse, Beckman Coulter), anti-human TCRαβ (APC, mouse, BioLegend), anti-human CD8α (PE-Cy7, mouse, BD Pharmingen), and anti-human CD4 (APC-Cy7, mouse, BD Pharmingen).

### Phylogenetic and sequence conservation analysis

Sequences encoding proteasomal β subunit family proteins (IPR000243) were retrieved from InterPro (69), and 249 unique amino acid sequences were selected by applying a 90% sequence identity cutoff in CD-HIT (70). After sequence alignment with CLUSTAL Omega, evolutionary history was inferred by using the ML method with Le and Gacue (LG) amino acid replacement matrix. The ProtTest program (70) in MEGA X (71) was used to select the best-fit models of protein evolution for this alignment. The selection was scored on Akaike’s information content (70), which suggested the LG+G+I model. The bootstrap consensus tree inferred from 100 replicates was taken to represent the evolutionary history of the taxa analyzed. Branches corresponding to partitions reproduced in <50% of the bootstrap replicates were collapsed. Initial trees were obtained automatically by applying neighbor-joining and BioNJ algorithms to a matrix of pairwise distances estimated using the Jones-Taylor-Thornton (JTT) model and then selecting the topology with a superior log-likelihood value. Based on the best-fit model, a discrete gamma distribution was used to model evolutionary rate differences among sites (+G, parameter = 1.8638) and the rate variation model allowed for some sites to be evolutionarily invariable ([+I], 1.07% sites). This process yielded a total of 187 positions in the alignment for evolutionary analyses using MEGA X (71). Clade separation was done based on known protein sequences (or paralogs) in the PSMB family ML tree, and calculations of the position-wide residue conservation and LOGO plot were made using Geneious Prime (72). Multiple sequence alignment for the PSMB10 clade was used to map the residue conservation on the PSMB10 structure (chain H from PDB 6E5B) using ConSurf (73).

### Molecular modeling and MDSs

The PSMB10 structure (chain H from PDB 6E5B) was used to generate an in silico model for PSMB10 G209R. For modeling procedures and MDSs, only the globular domain of the protein (residues 40–230) was used, with the propeptide (residues 1–39) and an extended C-terminal region (residues 231–260) both removed as these regions are likely highly dynamic in the free protein and stabilized by extensive interactions within the assembled immunoproteasome. Corresponding structures of PSMB10 WT and PSMB10 G209R were prepared, and MDS was performed using the Protein Preparation Wizard and Desmond with OPLS3e force field in Schrödinger software (Schrödinger), respectively. Each system was first neutralized by adding sodium ions around the protein using the Schrödinger software System Builder module. The neutralized protein was placed in TIP3P water, and random water molecules were substituted to obtain an ionic strength of 150 mM. Each solvated system was next relaxed using a series of restrained minimization stages of 1-ns duration each: (1) all heavy atoms with a force constant of 1,000 kcal/molÅ^2^; all protein backbone atoms with a force constant of (2) 100 kcal/molÅ^2^ and (3) 5 kcal molÅ^2^; and finally, (4) with no restraints. Unrestrained MDSs were then performed for a total 120 ns, comprising a 20-ns equilibration period (excluded from subsequent analyses) and a 100-ns production run in the isothermal–isobaric ensemble using Langevin thermostat and barostat with relaxation times of 1 and 2 ps, respectively. The equations of motion were integrated using multiple time steps for short-range (2 fs) and long-range (6 fs) interactions with a 10 Å cutoff applied for nonbonded interactions. RMSD and RMSF values were output directly from Schrödinger software for each protein’s production run and plotted in GraphPad Prism 9. Difference RMSF (PSMB10 G209R minus PSMB10 WT) was calculated, and most significant differences were based on a ±2σ cutoff.

For each MD production run, representative structures of PSMB10 WT or PSMB10 G209R were selected within each 10-ns segment of the 100-ns simulation by identifying the structure with the lowest RMSD compared with the average structure within each interval. For protein–protein docking analysis, these 10 representative structures for each protein were individually docked with an assembly of all PSMB10 neighboring proteins in the immunoproteasome (i.e., PSMB1/2/3/4/6/9 from PDB 6E5B), using an Impref minimization in Schrödinger software allowing flexibility for all components. Potential energy of each docked complex was scored using the OPLS3e force field, and the resulting values were plotted in GraphPad Prism 9. Statistical analysis was accomplished in Prism using an unpaired *t* test (**, P < 0.002).

### Cell culture

Skin fibroblasts obtained from the patient (PSMB10 G209R) and a healthy control of comparative age (PSMB10 WT) were cultured in cell culture plates coated with 50 μg/ml PureCol (Advanced BioMatrix). The medium for cultivation comprised DMEM, high glucose (4.5 g/L), GlutaMAX (Thermo Fisher Scientific) supplemented with 10% (vol/vol) fetal bovine serum (Capricorn Scientific), and 100 U/ml penicillin/streptomycin (Gibco, Thermo Fisher Scientific). Fibroblasts were grown in a humidified environment with 5% CO_2_ at 37°C and passaged into a new cell culture plate twice a week when reaching confluency of 80–90% by trypsinization.

### IFN treatment

Recombinant human IFNγ (PeproTech) or recombinant human IFNβ (PeproTech) was dissolved in PBS, aliquoted, and stored at −80°C until further use. 200,000 cells were seeded in 6-well plates the day before treatment. Upon reaching 70–80% confluency, the cell culture medium was exchanged to medium with or without 75 U/ml recombinant human IFNγ or 100 U/ml recombinant human IFNβ for another 24 h. For MS analysis, 1 × 10^6^ cells were seeded in 150-mm plates and treated with IFNγ for 4 days, washed with PBS three times, harvested, and stored at −80°C until used.

### RNA isolation and quantitative real-time PCR (RT-qPCR) analysis

Cells were washed with PBS and detached with trypsin–EDTA (Gibco, Thermo Fisher Scientific) for 5 min at 37°C. Cell culture medium (containing 10% FCS) was used to stop trypsinization. Cells were collected by centrifugation for 5 min at 1,500 rpm at room temperature. Cell pellets for protein extraction were stored at −80°C. Cell pellets for RNA extraction were directly lysed in 1 ml TRIzol Reagent (Invitrogen) and stored at −80°C until RNA isolation. For that, 0.2 ml chloroform was added to the TRIzol lysed sample and the mixture was vigorously shaken, incubated for 3 min, and then centrifuged at 14,000 rpm at 4°C for 5 min. The upper aqueous phase was transferred into a new tube, and 0.5 ml isopropanol was added. The samples were incubated for 10 min and centrifuged at 14,000 rpm at 4°C for 10 min. The supernatant was then discarded, and the pellet was washed in 1 ml of 75% ethanol and air-dried for 10 min. RNA was dissolved in RNase-free water.

Reverse transcription of 1 µg RNA into cDNA was performed using Maxima First Strand cDNA Synthesis Kit (K1641; Thermo Fisher Scientific). 2.5 μl of diluted cDNA samples was mixed with 5 µl LC480 SYBR Green I Master Mix (Roche) and 2.5 μl of forward and reverse primer (final concentration of 0.5 pmol/μl) dilutions. RT-qPCR was performed using a LightCycler 480 instrument (Roche Diagnostic). Primers used are listed in Table 2.

**Table 2.** Primers used

Gene	Forward (5′-3′)	Reverse (5′-3′)
*RPL19*	5′-TGT​ACC​TGA​AGG​TGA​AGG​GG-3′	5′-GCG​TGC​TTC​CTT​GGT​CTT​AG-3′
*PSMA3*	5′-AGA​TGG​TGT​TGT​CTT​TGG​GG-3′	5′-AAC​GAG​CAT​CTG​CCA​ACA​A-3′
*PSMB5*	5′-TCA​GTG​ATG​GTC​TGA​GCC​TG-3′	5′-CCA​TGG​TGC​CTA​GCA​GGT​AT-3′
*PSMB6*	5′-CAG​AAC​AAC​CAC​TGG​GTC​CT-3′	5′-CCC​GGT​ATC​GGT​AAC​ACA​TC-3′
*PSMB7*	5′-TCG​CTG​GGG​TGG​TCT​ATA​AG-3′	5′-TCC​CAG​CAC​CAC​AAC​AAT​AA-3′
*PSMB8*	5′-GCT​ATT​CTG​GAG​GCG​TTG​TC-3′	5′-AGG​CCT​CTT​CTT​CTC​CTT​GG-3′
*PSMB9*	5′-ATG​CTG​ACT​CGA​CAG​CCT​TT-3′	5′-GCA​ATA​GCG​TCT​GTG​GTG​AA-3′
*PSMB10*	5′-AGC​CCG​TGA​AGA​GGT​CTG​G-3′	5′-CAT​AGC​CTG​CAC​AGT​TTC​CTC​C-3′

### Protein extraction and BCA assay

Cell pellets were lysed in OK40 buffer (50 mM Tris-HCl, pH 7.5, 2 mM DTT, 5 mM MgCl_2_, 10% glycerol, 2 mM ATP, 0.5% NP-40) supplemented with protease inhibitor cocktail (Roche) and phosphatase inhibitor cocktail (Roche) for 20 min on ice. Protein lysates were collected after being centrifuged at 14,000 rpm for 20 min at 4°C. Protein concentrations were determined using Pierce BCA Protein Assay Kit (Thermo Fisher Scientific) according to the manufacturer’s instructions. Bovine serum albumin diluted to a range from 0 to 2 μg/μl in PBS served as standard samples. Samples or empty lysis buffer was diluted 1:10 in PBS to a total volume of 20 μl and mixed with 200 μl BCA working solution. After 30-min incubation at 37°C, absorbance was measured at 562 nm using a Sunrise Absorbance microplate reader (TECAN).

### Western blotting

SDS-PAGE was performed using standard protocols as described before (74). Gels were blotted onto polyvinylidene difluoride membranes at 250 mA for 90 min on ice. The membranes were then incubated for 1 h at room temperature with Roti-Block (Carl Roth) for blocking unspecific protein interactions. Membranes were incubated with primary antibodies (usually 1:1,000 dilutions) at 4°C overnight, washed three times with PBS, 0.1% Tween-20 (PBST; AppliChem), and further incubated with secondary antibodies at room temperature for 2 h (1:20,000 dilution). After washing with PBST (3 times), membranes were incubated with 1 ml of chemiluminescent substrates (SuperSignal West Pico PLUS Kit, Thermo Fisher Scientific). Detection of chemiluminescence and analysis of signal intensity were done with iBright FL1500 Imaging System (Thermo Fisher Scientific). The following primary antibodies were used: anti-LMP2 (ab3328; Abcam), anti-LMP7 (ab3329; Abcam), anti-MECL1 (ab183506; Abcam), anti-α1-7 (ab22674; Abcam), anti-ß1 (MCP421; Enzo Life Sciences), anti-ß2 (MCP168; Enzo Life Sciences), anti-ß5 (ab90867; Abcam), anti-β-actin (A3854; Sigma-Aldrich), anti-mouse IgG HRP-linked (#7076; Cell Signaling Technology), and anti-rabbit IgG HRP-linked (#7074; Cell Signaling Technology).

### ABP proteasome activity analyses

Activity of catalytic subunits PSMB6/PSMB9, PSMB7/PSMB10, and PSMB5/PSMB8 was assayed in native protein extracts using ABPs kindly provided by Bobby Florea and Hermen Overkleeft Leiden Institute of Chemistry, Leiden University, Einsteinweg 55, 2333 CC, Leiden, the Netherlands (27). 20 μg of protein was incubated either with 0.5 μM MV151 (pan-ABP) or with a mixture of 0.25 μM LW124 (PSMB6/PSMB9 specific ABP) and 1 μM MVB127 (PSMB5/PSMB8 specific ABP), respectively, at 37°C for 1 h. Protein samples were then boiled at 98°C for 5 min after mixing with 6× Laemmli buffer (1 M Tris-HCl, pH 6.8, 15% glycerol, 6% SDS, 1% bromophenol blue). 15% Tris-glycine SDS–polyacrylamide gels were used for protein separation. Samples and protein marker (A8889; AppliChem) were loaded onto the gel, and gels were first run for 30 min at 90 V and then at 120 V for 2 h. Fluorescence signals of covalently labeled catalytic proteasome subunits were detected and analyzed using iBright FL1500 Imaging System with the Cy3 channel for MV151 and MVB127 and the Cy2 channel for LW124.

### Transcriptome analysis

Total RNA from PSMB10 WT and PSMB10 G209R skin fibroblast was extracted using TRIzol and quality-controlled by Bioanalyzer (Agilent Technologies) using the RNA 6000 Nano Kit according to the manufacturer’s instructions. RNA that passed quality control assessment was forwarded to reverse transcription, amplification, Cy3 labeling, and subsequent hybridization to the Human Gene Expression Microarray 4 × 44 k v2 (Agilent) using Low-Input Quick Amp Labeling Kit (Agilent Technologies) as described before (75).

Raw microarray data were imported into GeneSpring software v 14.9 (Agilent), compromised probes were removed from further analyses, and the data were subsequently log2-transformed and normalized using Percentile Shift. A one-way ANOVA with Tukey’s honestly significant difference post hoc testing and Benjamini–Hochberg multiple testing correction was calculated to retrieve DEGs between the experimental conditions (PSMB10 WT and PSMB10 G209R, untreated versus IFNγ-treated). Condition-specific gene sets were assessed by overlaying different lists of DEGs from Tukey’s post hoc testing via Venn diagrams and selecting unique DEGs. DEGs identified from the microarray analysis were subjected to GO pathway enrichment analysis using the clusterProfiler R package 4.12.6 (76). The gene set of interest was defined based on adjusted P values <0.05 and |log2fold change| >2.

Molecular Signatures Database was used as reference gene sets related to IFNs. Gene set enrichment analysis was conducted using clusterProfiler R package 4.12.6 to assess pathway enrichment in DEGs between PSMB10 G209R and PSMB10 WT. Data visualization including Venn diagrams and heatmaps was performed using R 4.2.0 and an online platform for data analysis and visualization.

### Proteasome immunopurification and MS analysis

This assay was done as previously described (29). Briefly, 200 μg of mouse IgG_1_ monoclonal anti-α2 antibody MCP21 was cross-linked onto 200 μl 25% slurry of Protein G MagBeads (GenScript). The purification was done at 4°C. Cells were lysed with lysis buffer (pH 7.6, 20 mM Tris-HCl, 0.25% Triton X-100, 100 mM NaCl, 10 mM EDTA, 10 mM ATP, 5 mM MgCl_2_) and 1 tablet of protease and phosphatase inhibitors per 50 ml (cOmplete ULTRA Tablets Mini EDTA-free and PhosSTOP, Roche), sonicated (Bioruptor Plus, Diagenode; 15 cycles, 45 s on and 15 s off), and then centrifuged at 16,000 × *g* for 30 min. The supernatant with at least 2–3 mg of total protein was then mixed with the MagBeads and incubated overnight. The next day, the beads were washed with equilibration buffer (20 mM Tris-HCl, 1 mM EDTA, 10% glycerol, 100 mM NaCl, 2 mM ATP, 5 mM MgCl_2_, pH 7.6). Three fourths of the beads were eluted with equilibration buffer supplemented with 3 M NaCl for TD-MS, while the remaining fourth was eluted with 5% SDS in 50 mM ammonium bicarbonate, pH 7.55, for BU-MS. The eluate for TD-MS was concentrated and buffer-exchanged by ultrafiltration molecular weight cut-off ([MWCO] 100 kDa) with 200 mM ammonium acetate, pH 7.4. The sample was then separated on a nanoLC system using a reversed-phase C4 precolumn (PepMap 300 C4 5 μm, 300 μm i.d. × 5 mm, Thermo Fisher Scientific) and ReproSil-Pur C4 column (3 μm, 75 μm i.d. × 15 cm; packed in-house) and analyzed using an Orbitrap Fusion Tribrid mass spectrometer (Thermo Fisher Scientific) in intact protein mode as described previously (29). Mass spectra were deconvoluted and visualized with RoWinPro and VisioProtMS (77). The eluate for BU-MS was reduced with 100 mM Tris(2-carboxyethyl)phosphine and alkylated with 400 mM 2-chloroacetamide at 95°C for 5 min. Each sample was loaded on an S-trap spin column (ProtiFi), according to the manufacturer’s instructions and digested with trypsin (Promega) overnight at 37°C. Digested peptide extracts were analyzed by online nanoLC using a reversed-phase C18 precolumn (PepMap 100 C18 5 μm, 100 Å, 300 μm i.d. × 5 mm, Thermo Fisher Scientific) and ReproSil-Pur C18-AQ column (3 μm resin, 75 μm i.d. × 50 cm; packed in-house) coupled to an Orbitrap Fusion Tribrid mass spectrometer operating in positive mode (29). Peptide and protein identification was done with Mascot database search engine using the UniProt human proteome including 20S variants and contaminants databases. Validation and label-free quantification were done using Proline (78), where peptides along the G209R mutation were also confirmed. Protein quantification was done using only specific peptides, and abundance was attained by median ratio fitting based on quantified peptide ions. Normalization was done by the total abundance, and missing value inference was applied using 5% noise. Intensity-based absolute quantification was then calculated by dividing the intensities by the number of theoretically observable tryptic peptides (79). Finally, the quantification of stoichiometries was done as previously described (80).

### Substrate-based proteasome activity assay

The activity assay was performed in 96-well black plates (Greiner Bio-One). 10 μl of clear lysate (corresponding to 10 µg total proteins) was diluted with 40 μl of 100 mM Tris-HCl, pH 8, buffer. Then, 50 μl of 100 µM fluorogenic peptide substrate was added: Suc-LLVY-AMC, Boc-LRR-AMC, or z-LLE-AMC, to probe for chymotrypsin-, trypsin-, or caspase-like activities, respectively, was added. The fluorescence intensities at λEx/λEm of 360/460 nm and at 37°C were measured in a microplate fluorimeter (CLARIOstar, BMG Labtech) for 12 cycles, with one reading every 5 min. The slope of the increase in fluorescence intensity over time (within linear range) was calculated, and normalization was done using the average slope for the controls. Mean and standard deviation were calculated from four replicates. Statistical difference was determined based on the two-tailed P value from an unpaired *t* test (Student’s *t* test).

### Online supplemental material

Fig. S1 shows RNA and protein expression analysis of immuno- and standard proteasome subunits in PSMB10 WT and PSMB10 G209R skin fibroblasts. Fig. S2 presents GO and pathway enrichment analyses of DEGs following IFNγ stimulation. Fig. S3 illustrates ex vivo T cell differentiation from patient-derived and control HSCs in ATO cultures. Fig. S4 demonstrates normal phenotype and functional responses of PSMB10 G209R T cells. Fig. S5 summarizes bulk TCR and BCR repertoire sequencing metrics and gene usage analysis.

## Supplementary Material

SourceData F4 is the source file for Fig. 4.

SourceData FS1 is the source file for Fig. S1.

## Data Availability

The data generated in this study have been made publicly available in Gene Expression Omnibus under GSE288134 (GEO, RRID:SCR_005012).
