# Supplementary figures and images for: Dominant-negative PSMB10 disrupts immunoproteasome assembly and leads to transient T lymphopenia
Source: J Hum Immun. 2026 Apr 9;2(3):e20250129. doi: 10.70962/jhi.20250129 (PMC13064613; doi:10.70962/jhi.20250129)

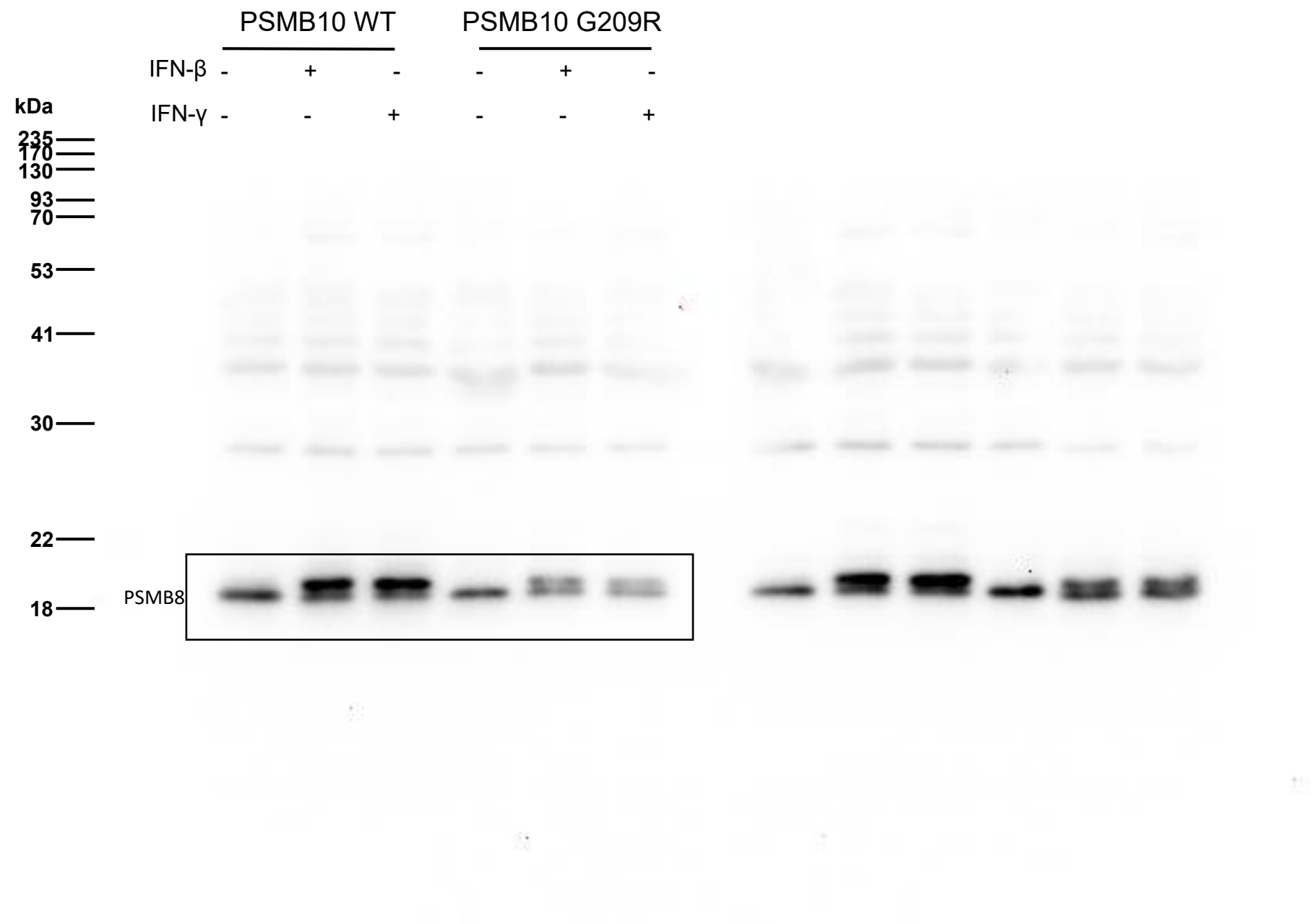

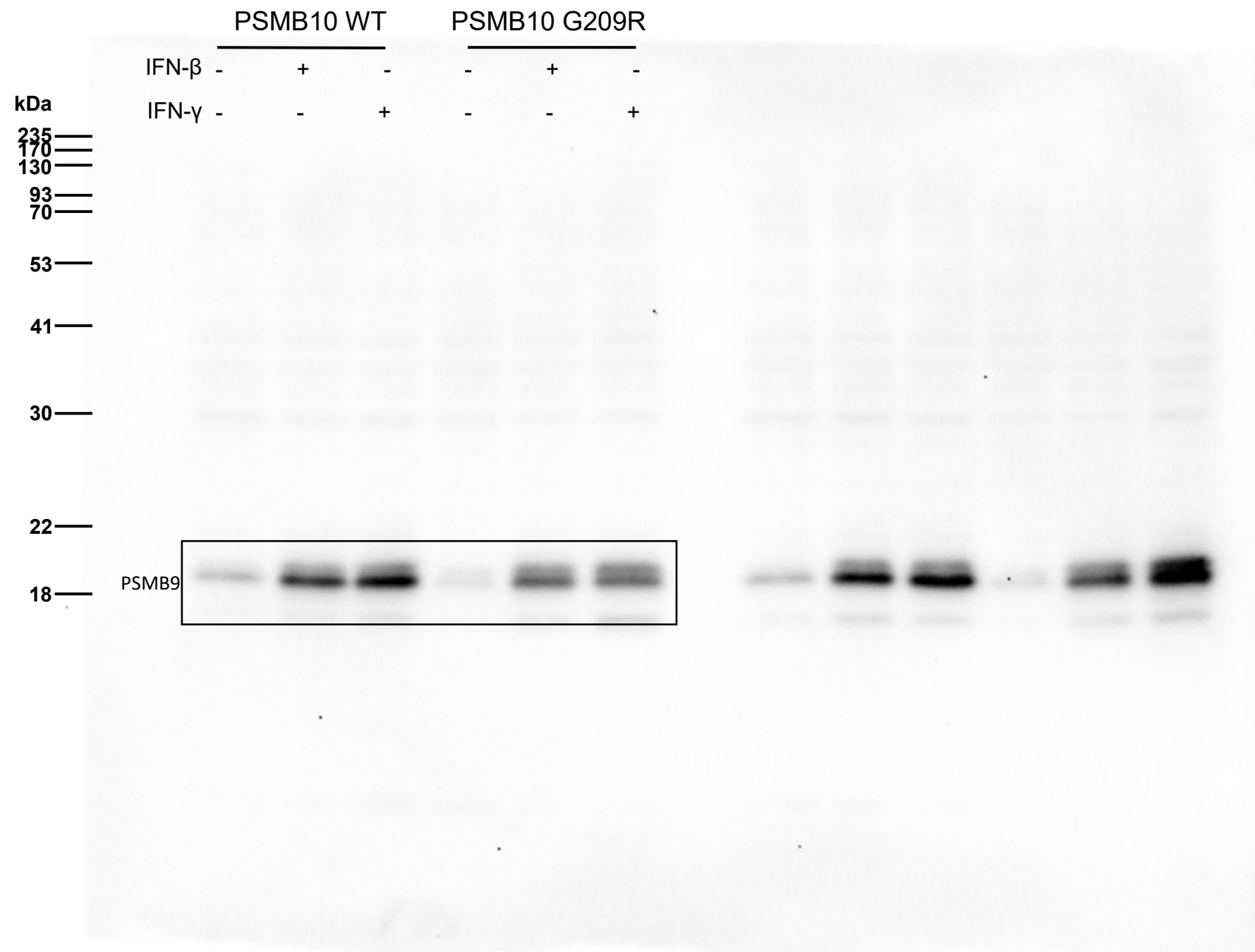

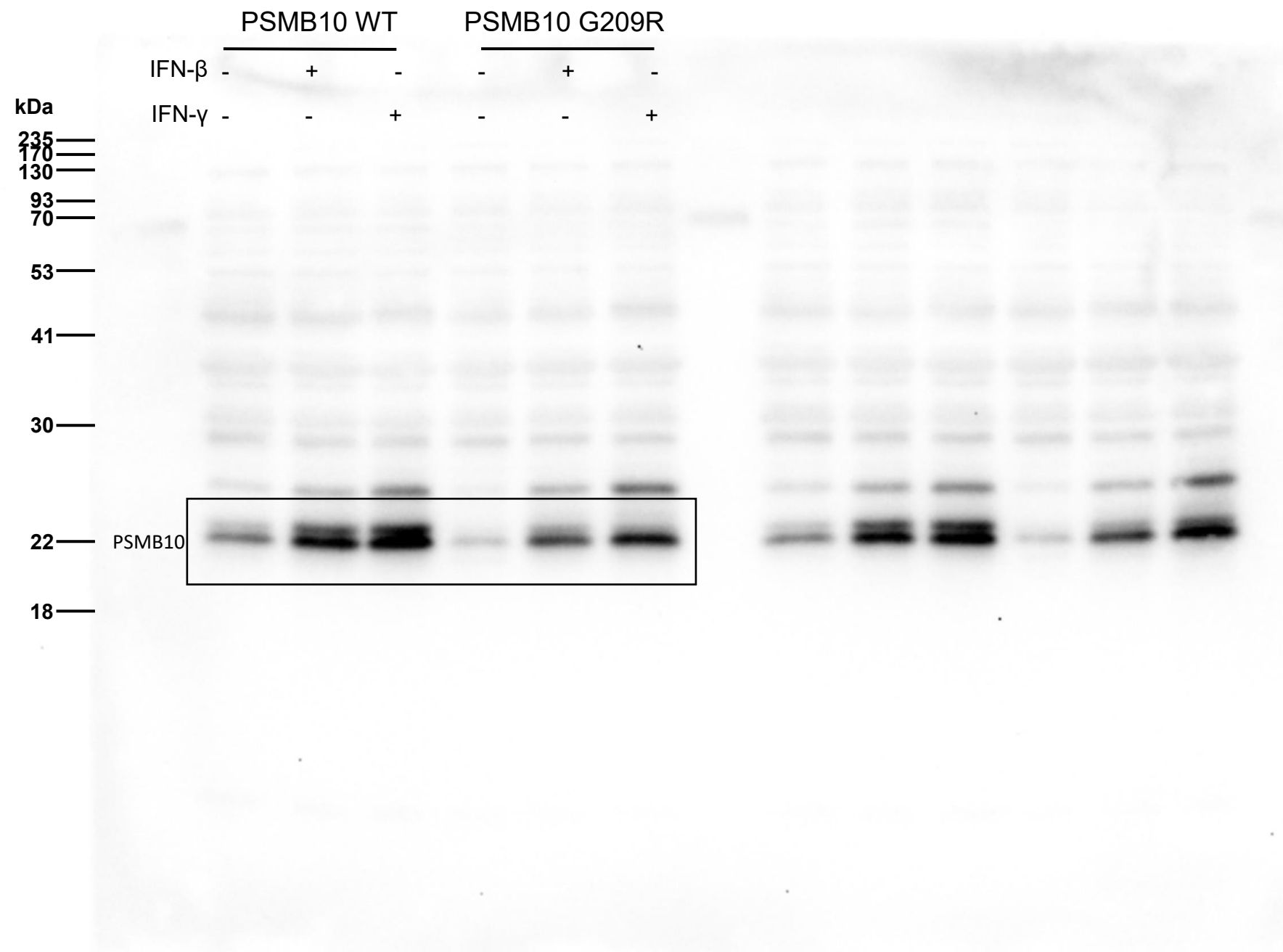

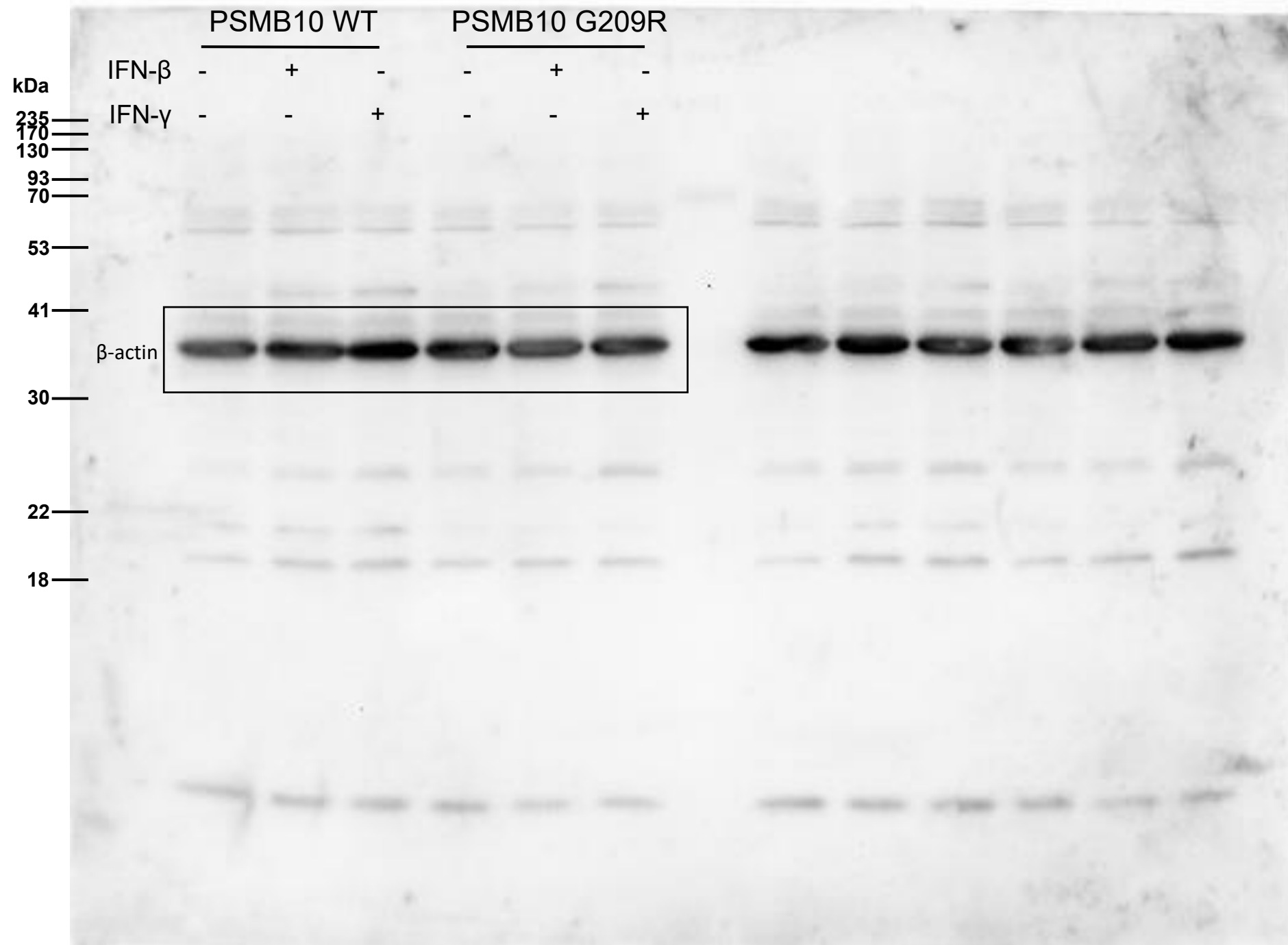

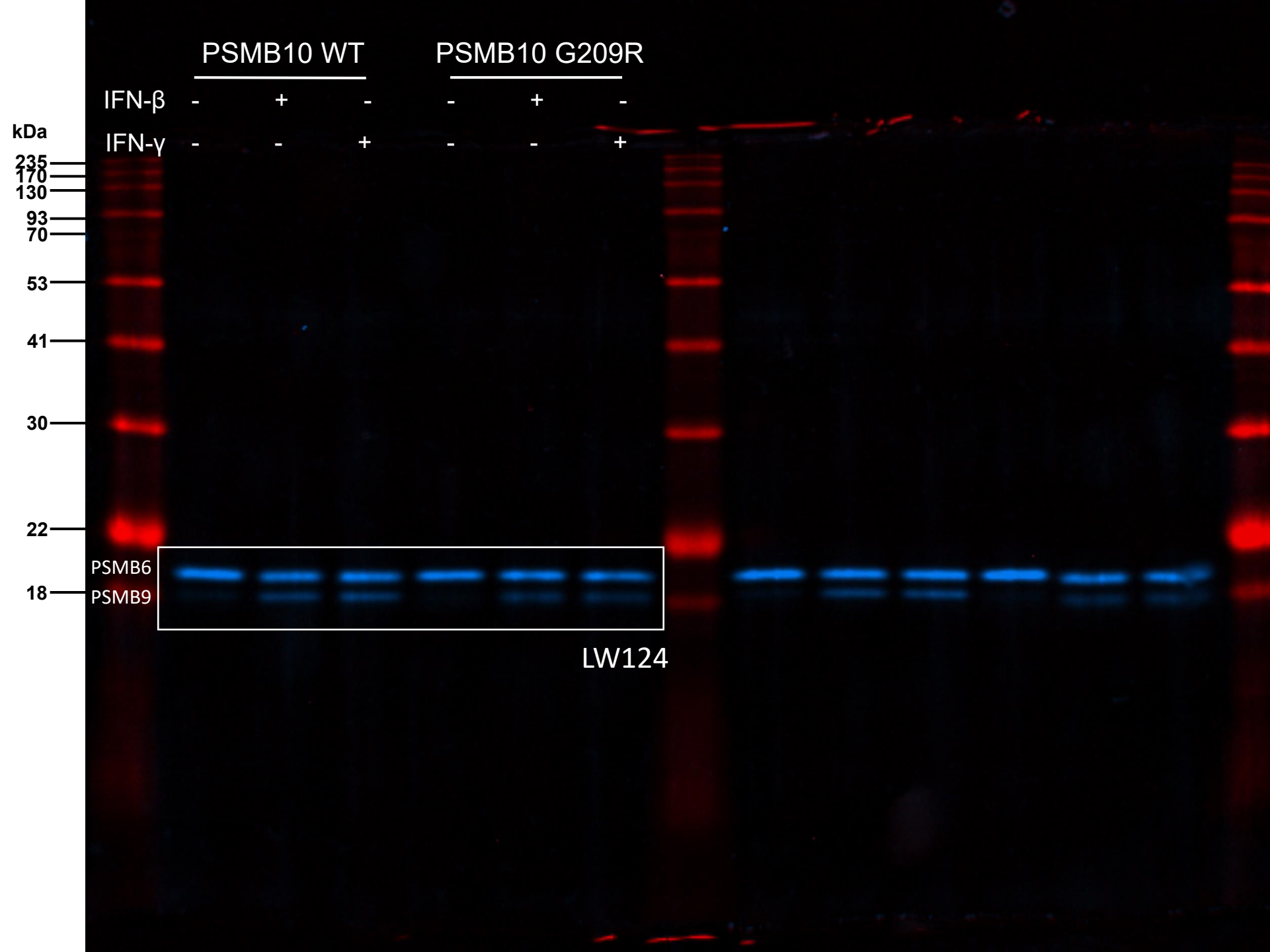

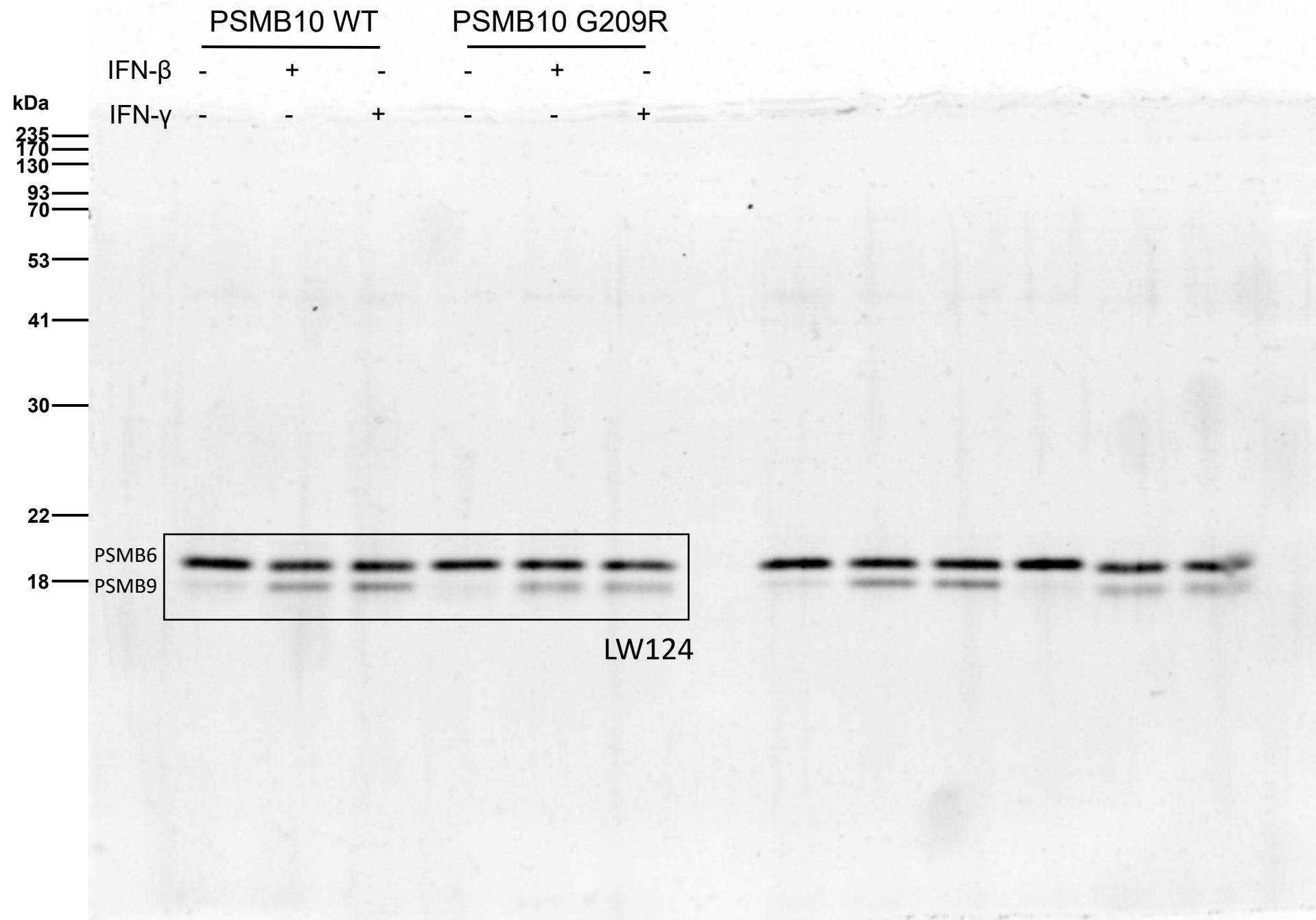

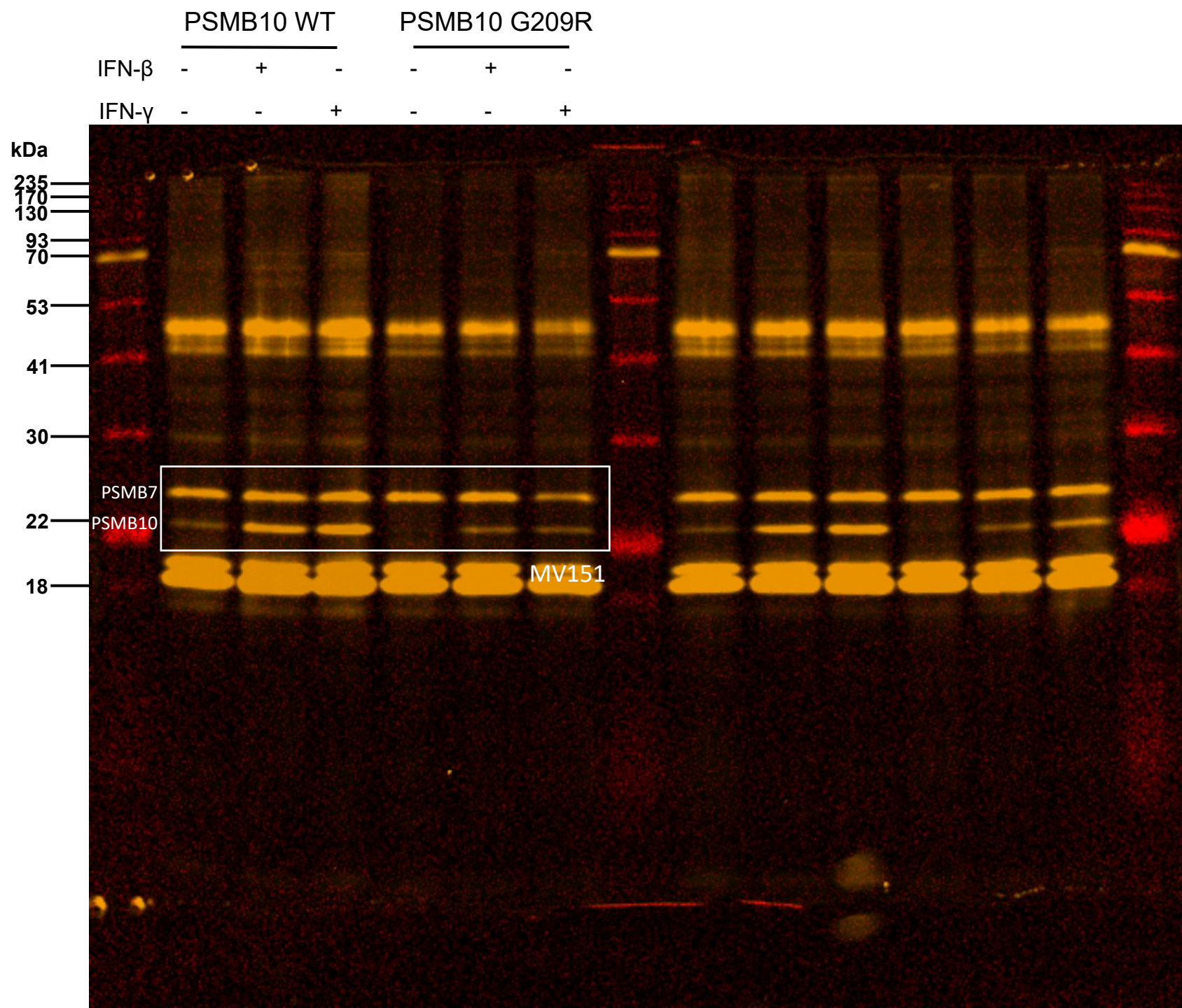

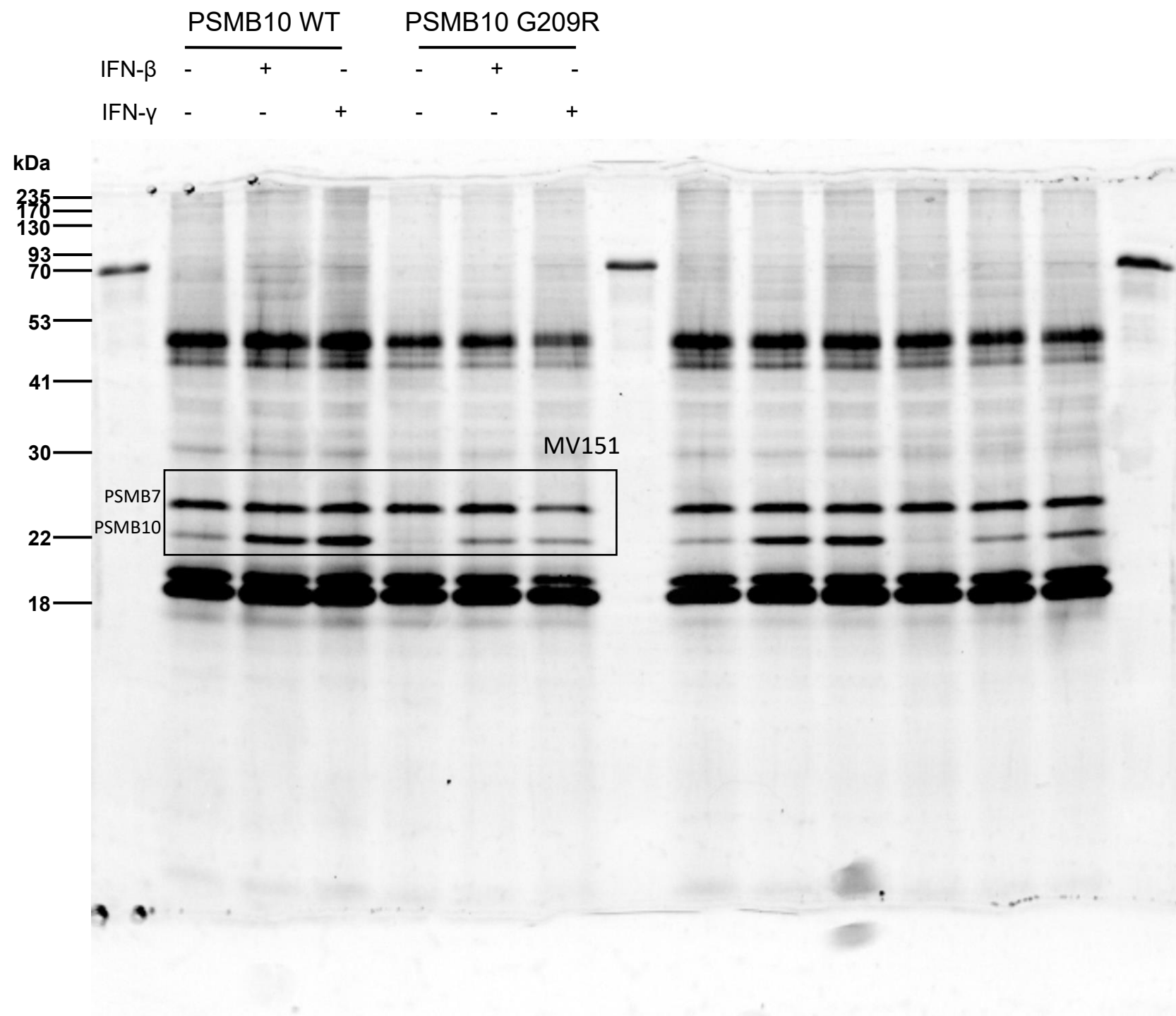

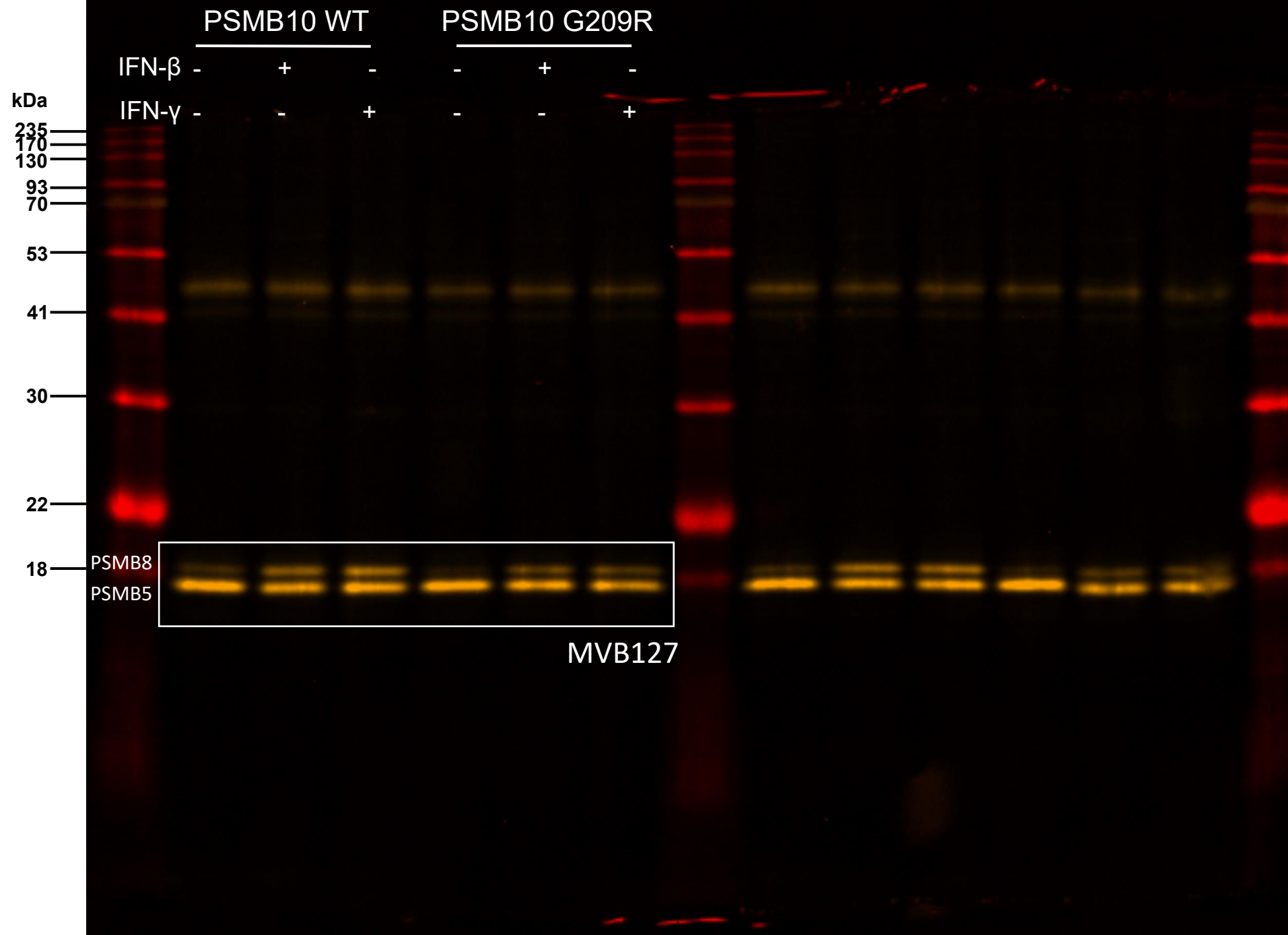

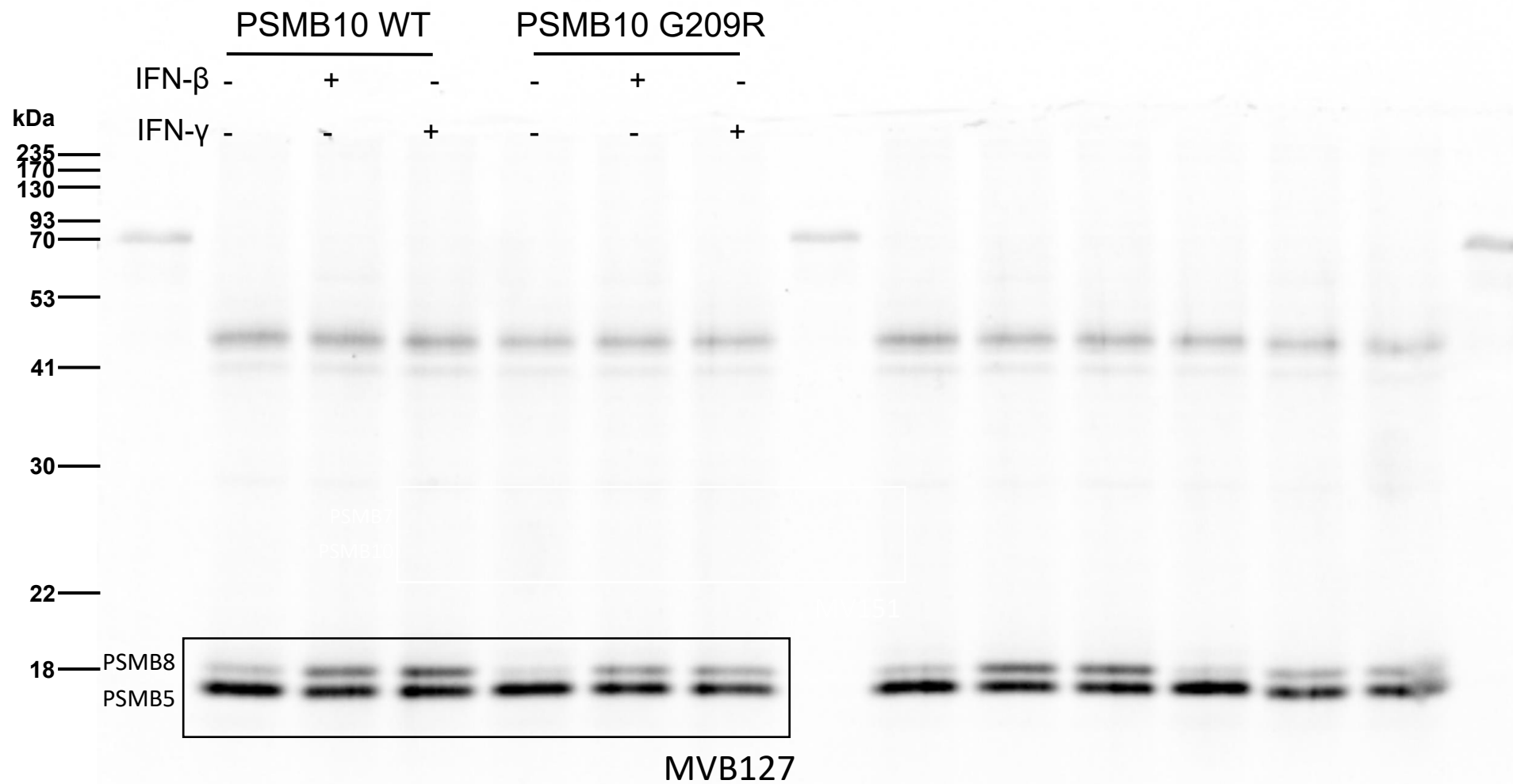

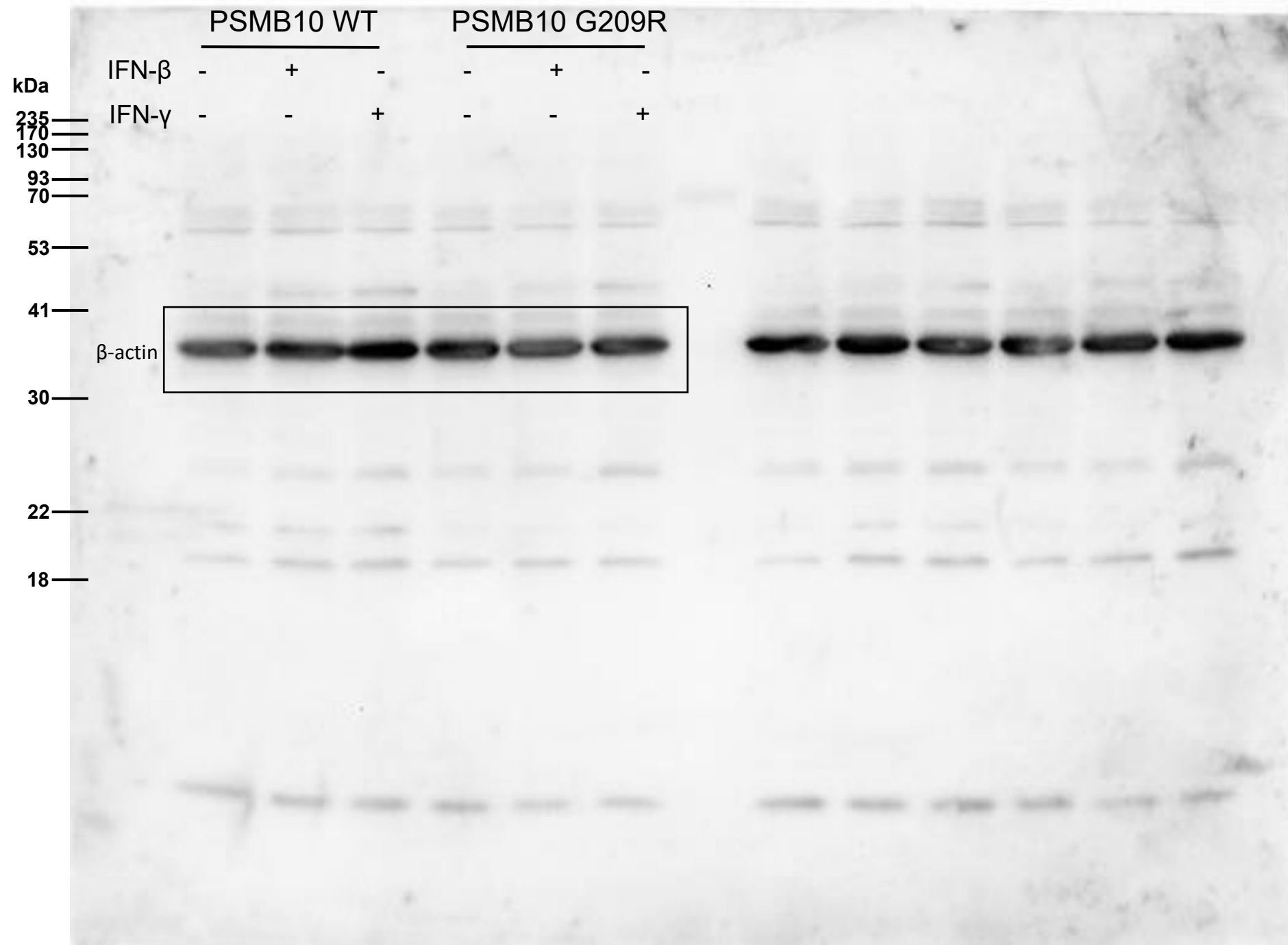

Supplement: SourceData F4 — is the source file for Fig. 4. [file jhi_20250129_sourcedataf4.pdf]

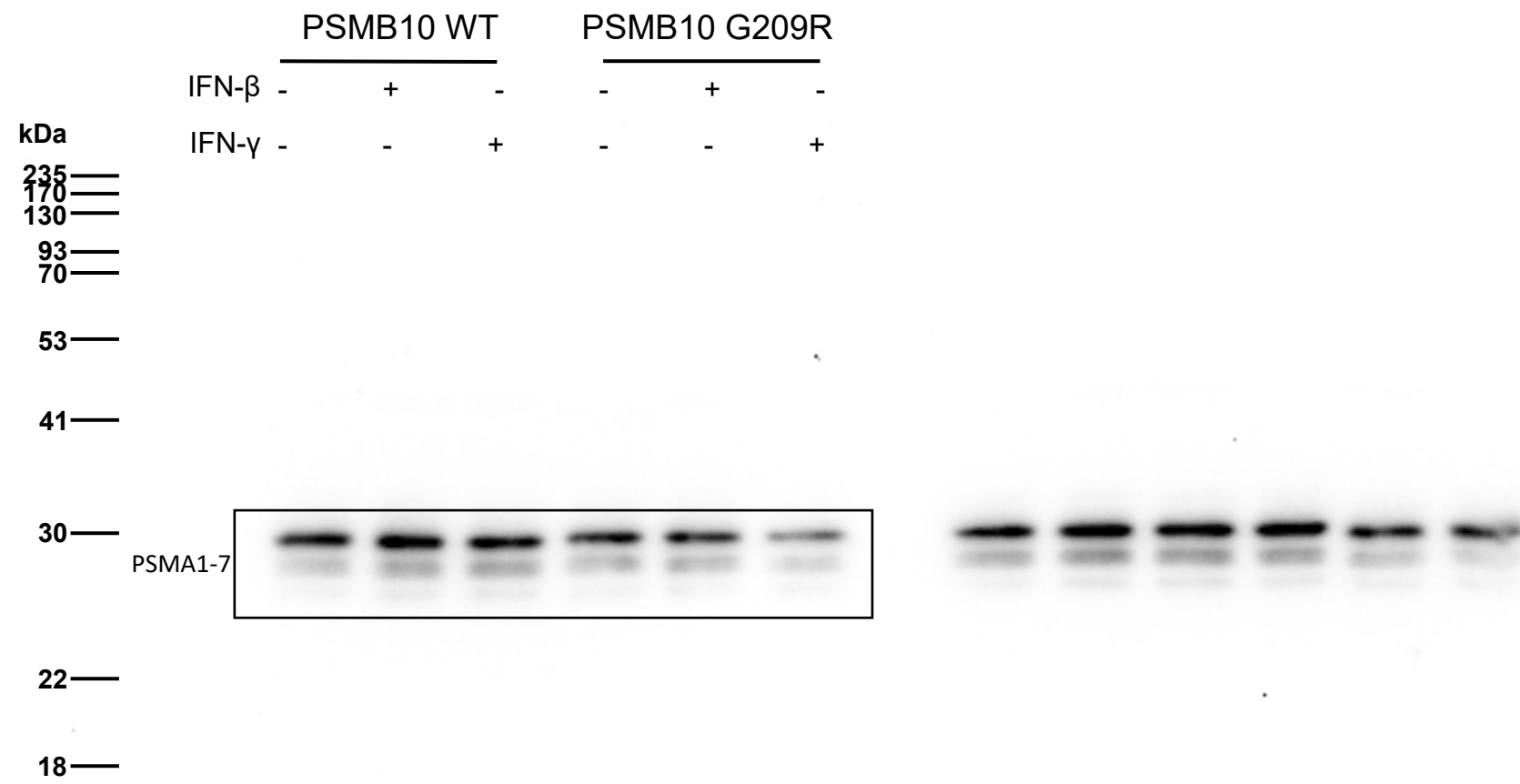

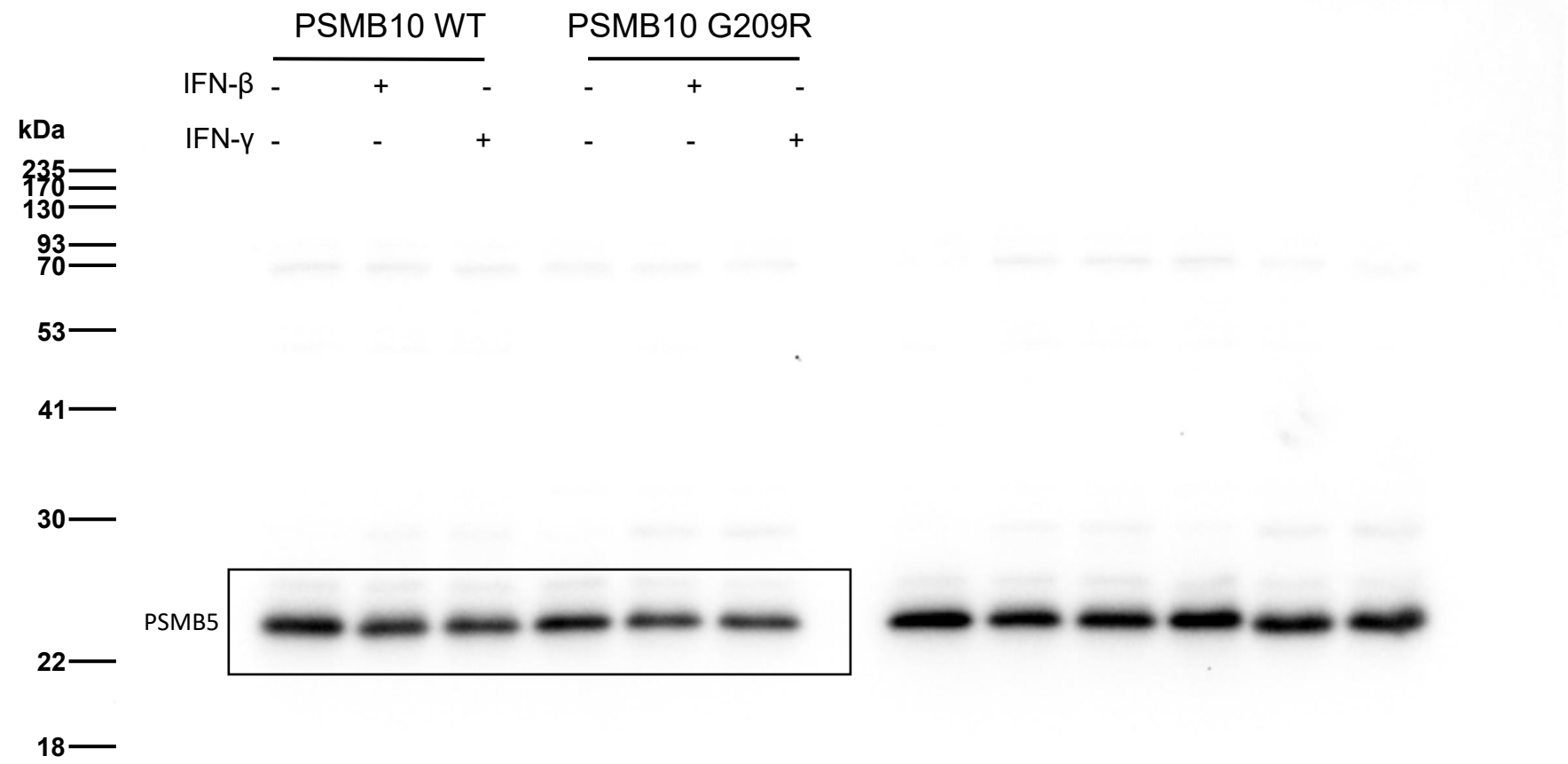

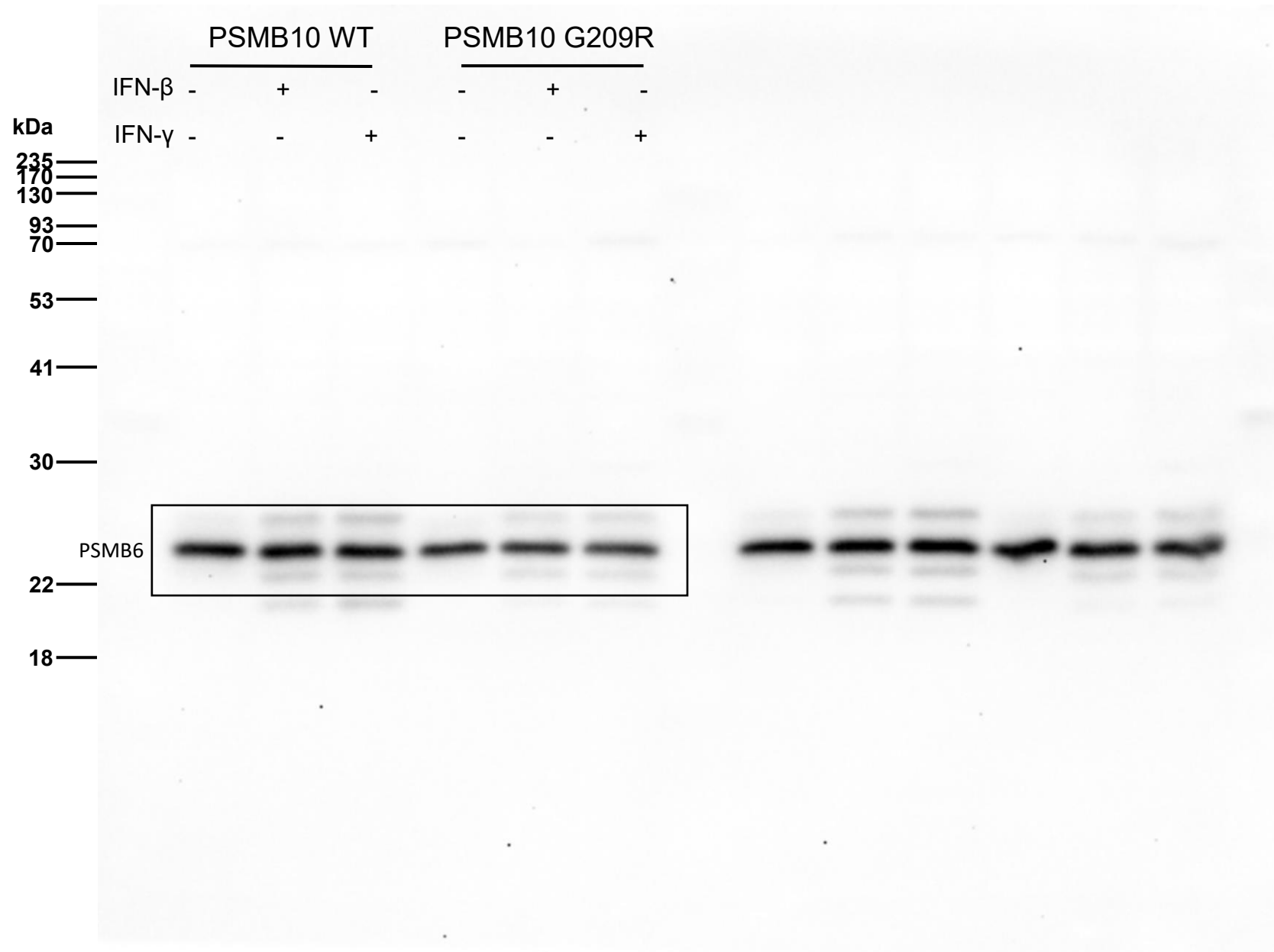

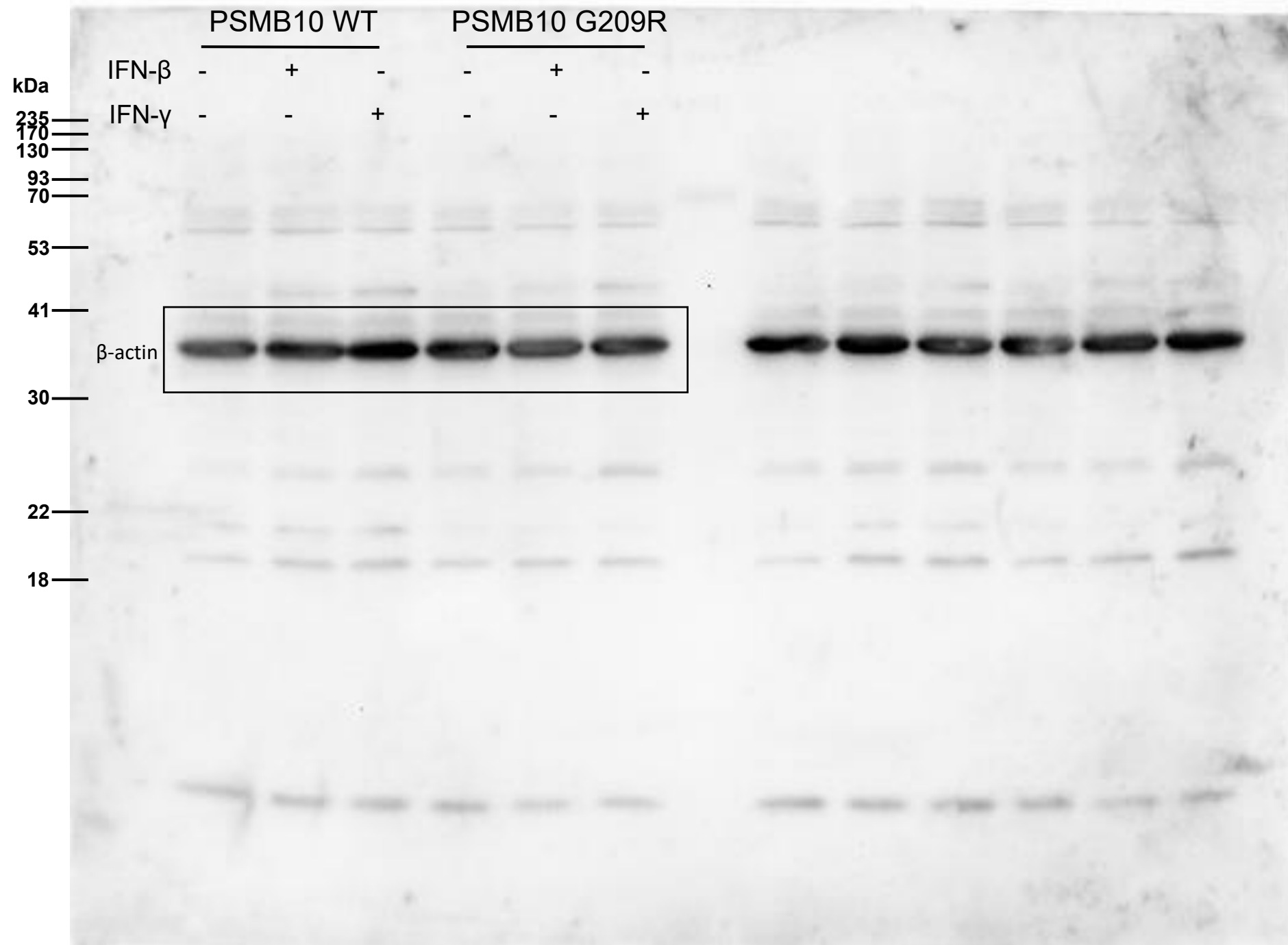

Supplement: SourceData FS1 — is the source file for Fig. S1. [file jhi_20250129_sourcedatafs1.pdf]
